# Investigating the Therapeutic Efficacy of Quality-Controlled, miR-146a-5p-Enriched Small Extracellular Vesicles Derived From MSCs Against Idiopathic Pulmonary Fibrosis

**DOI:** 10.1007/s12015-025-10976-8

**Published:** 2025-09-24

**Authors:** Xin Wang, Lingjiao Meng, Qiuhong Wang, Ruixue Rong, Yu Zhang, Xiaohui Zhao, Chen Liang, Huizhen Guo, Li Deng, Zengqi Tan, Feng Guan, Yi Tan

**Affiliations:** 1 R&D Department, Shandong Qilu Cell Therapy Engineering Technology Co., Ltd., Gangyuan 6th Road, Licheng District, Ji’nan, Shandong 250000 P. R. China; 2 Shandong Yinfeng Life Science Research Institute, Ji’nan, P. R. China; 3 Department of Otolaryngology, Head and Neck Surgery, Yantai Yuhuangding Hospital, Qingdao University, Yantai, P. R. China; 4 Department of Gastroenterology, The First Affiliated Hospital of Shandong First Medical University & Shandong Provincial Qianfoshan Hospital, Ji’nan, P. R. China; 5 School of Medicine, Northwest University, Xi’an, P. R. China; 6 Key Laboratory of Resource Biology and Biotechnology in Western China, Ministry of Education, Provincial Key Laboratory of Biotecnology, College of Life Sciences, Northwest University, Xi’an, P. R. China

**Keywords:** Umbilical cord mesenchymal stem cell, Quality control strategy, Key therapeutic molecules, Nebulization

## Abstract

Small extracellular vesicles derived from umbilical cord mesenchymal stem cells (UC-sEvs) may be used for the treatment of idiopathic pulmonary fibrosis (IPF) because of their ability to control inflammation and inhibit fibrosis. However, the lack of clarity regarding the treatment mechanism of IPF and the corresponding quality standards limit the clinical application of these small extracellular vesicles. Here, we established a good manufacturing practice (GMP) grade process for isolating UC-sEvs, and RNA-seq was performed to screen for potential therapeutic cargo in the product to confirm the therapeutic effect of nebulized UC-sEv agents against IPF. Functionally, UC-sEvs inhibited the pulmonary inflammatory response by regulating macrophage function, thereby suppressing the bleomycin toxicity-induced progression of fibrosis. Mechanistically, miR-146a-5p enrichment in UC-sEvs may be involved in alleviating bleomycin-induced IPF by targeting TRAF6/IRAK1 to negatively regulate inflammation. The proposed quality control strategy ensures the stability of the product across three batches, with RNA-seq analysis revealing highly similar miRNA expression profiles. The feasibility of using miR-146a-5p as a key therapeutic molecule has been validated. Finally, on the basis of the results of pharmacodynamics and key therapeutic molecule studies, we provided a detailed quality control standard for IPF therapy by nebulizing UC-sEv. These findings help understand how sEvs impact IPF and the possible consequences of their therapeutic usage and offer a quality standard reference.

**Graphical Abstract:**

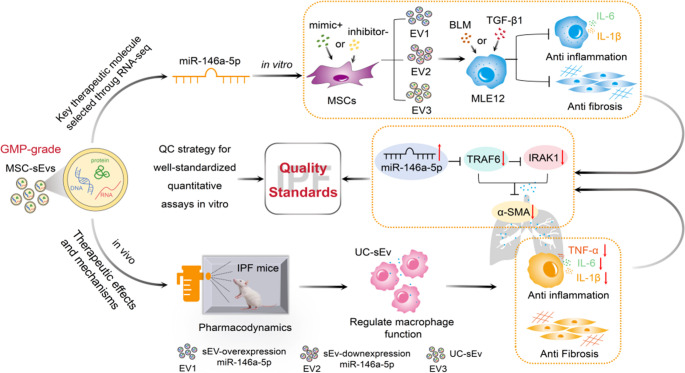

**Supplementary Information:**

The online version contains supplementary material available at 10.1007/s12015-025-10976-8.

## Introduction

Idiopathic pulmonary fibrosis (IPF) is a rare chronic fibrotic interstitial lung disease [1]. It is characterized by the progressive and uncontrollable destruction of oxygen exchange surfaces and airways and has become one of the primary causes of death worldwide. Because the etiology remains unknown and effective treatment methods are lacking, only a few treatment options are available for IPF, and the median period of survival from diagnosis is only 3–5 years [2, 3]. Currently, no effective treatment other than lung transplantation can completely reverse the disease [4], but challenges such as donor organ shortages, immune rejection, and surgical complications remain.

Mesenchymal stem cells (MSCs) are powerful candidates for regeneration in patients with lung injury and have been widely investigated in respiratory diseases [5, 6]. In addition, the benefits of MSCs are predominantly related to their paracrine factors, particularly small extracellular vesicles (sEvs) [7, 8]. sEvs include specific molecules, including microRNAs (miRNAs), growth factors, and intact fresh mitochondria, and thus can transport these molecules to the targeted site [9]. Specifically, miRNAs function as crucial regulators of almost all cellular activities, including immunoregulation [10, 11]. sEvs can adhere to and fuse with circulating or distal cells, which enables the captured sEvs to serve as shuttles for functional nucleotides or proteins and to horizontally transfer various biomolecules [12, 13]. sEvs derived from MSCs (MSC-sEvs) exert therapeutic effects on organ damage, including acute lung injury [14, 15]. MSC-sEvs are more popular in clinical applications, as many MSC therapy-related safety concerns [16], including (i) unexpected differentiation, (ii) tumor formation/promotion risks, (iii) emboli formation, (iv) entrapment in the lung microvasculature, and (v) immune rejection, might be avoided.

Despite MSC-sEv being therapeutically successful in preclinical studies, the current clinical applications of MSC-sEv are still associated with great challenges [17]. To investigate MSC-sEv as a therapeutic tool, several critical issues must be addressed, such as (i) rapid and reproducible GMP-grade manufacturing processes to meet clinical needs; (ii) identification of therapeutic molecules as a quality control point to ensure the effectiveness of IPF treatment; and (iii) implementation of a quality control strategy on the basis of IPF clinical efficacy and feasibility. Here, we discuss the aforementioned three issues. On the basis of our previous experience in the large-scale production and quality control (QC) of GMP-grade umbilical cord mesenchymal stem cells (UCMSCs) [18], we propose QC strategies for developing clinical-grade sEv derived from UCMSCs (UC-sEvs). Before initiating these clinical trials, sEv biological agents were examined in well-standardized quantitative assays in vitro or in appropriate animal models in vivo. Overall, we hope to offer ideas for the clinical translation and application of UC-sEvs through this study.

## Materials and Methods

### Cell Bank Construction and Identification of UCMSCs

UCMSCs were generated from the umbilical cord of a healthy human volunteer aged 29 years who gave birth at Yantai Yuhuangding Hospital (Yantai, China) on May 30, 2022, following the acquisition of written informed consent and ethical approval. Donor blood was subjected to virus testing, which included screenings for the hepatitis C virus (HCV), hepatitis B virus (HBV), human immunodeficiency virus (HIV), Treponema pallidum (TP), cytomegalovirus (CMV), human T-lymphotropic virus (HTLV), and Epstein Barr virus (EBV) were used, and all the results were negative for umbilical cord collection.

UCMSCs from GMP facilities were obtained and cultured as described previously [18]. In brief, UCMSCs were continuously cultured in basic MSC medium (Beijing Yocon Biology Co., Ltd., China) supplemented with serum-free medium (Beijing Yocon Biology Co., Ltd., China) at 37 °C in a 5% CO_2_ humidified atmosphere until approximately 80–90% confluence was attained. The cells were dissociated via trypsin/EDTA solution (Gibco, Thermo Fisher Scientific, USA), washed twice with Dulbecco’s phosphate-buffered saline (HyClone, USA), harvested via centrifugation for 5 min at 300 × *g*, and stored in a liquid nitrogen tank. Through successive passages, a master cell bank (MCB) was established after one generation (P1), followed by a postproduction cell bank (PPCB) after three generations (P3). All production stages strictly adhered to GMP principles, with careful attention to critical steps throughout each phase to ensure the final product’s expected quality.

UCMSCs were characterized for the expression of cell surface markers via flow cytometry (BD Biosciences, USA) on the basis of the following criteria: CD 105/CD 73/CD 90/CD 44 ≥ 95% and CD 34/CD 45/HLA-DR ≤ 2% (data not shown). Immunocytochemical staining of the cells was conducted with antibodies against CD 90 (cat. no. 328143, Biolegend, USA), CD 34 (cat. no. 343510, Biolegend, USA), CD105 (cat. no. 323208, Biolegend, USA), CD45 (cat. no. 304006, Biolegend, USA), CD 73 (cat. no. 344016; Biolegend, USA), CD 44 (cat. no. 338804; Biolegend, USA), and HLA-DR (cat. no. 307609; Biolegend, USA) conjugated with the fluorochrome phycoerythrin (PE), fluorescein isothiocyanate (FITC), and allophycocyanin (APC) (Biolegend, USA). The ability of UCMSCs to differentiate into adipogenic, osteogenic, and chondrogenic lineages was also evaluated as described in a previous study (data not shown) [18].

### Cell Culture and Macrophage Polarization

Human skin fibroblast (HSF) cells, human umbilical vein endothelial cells (HUVECs), and human leukemia monocytic cells (THP-1) were purchased from the National Collection of Authenticated Cell Cultures (Shanghai, China). Murine lung epithelial cells (MLE-12) were a gift from the First Affiliated Hospital of Naval Medical University (Department of Burn Surgery). The HSF cells and HUVECs were grown in Dulbecco’s modified Eagle medium (cat. no. c11995500BT; Gibco, Shanghai, China) supplemented with 10% or 15% fetal bovine serum (FBS, cat. no. C2830-0500, Vivacell, Shanghai, China). MLE-12 and THP-1 cells were grown in 1640 medium (cat. no. C11875500BT, Gibco, China) supplemented with 10% FBS. All cell types were grown in a monolayer at 37 °C in a humidified atmosphere of 5% CO_2_ and 95% air, collected at 90% confluence, and used for further analysis.

THP-1 cells (1 × 10^6^) were incubated with 100 ng/mL phorbol 12-myristate 13-acetate (PMA, cat. no. 50601ES03, Yeasen, China) for 24 h to induce their differentiation into M0 macrophages. The cells were continuously cultured with or without interferon gamma (IFN-γ, 10 ng/mL, cat. no. P5664, Beyotime, China), lipopolysaccharide (100 pg/mL, cat. no. L8880, Solarbio, China), or 5 × 10^9^ UC-sEv particles for 24 h. The prepared cells were immunostained with CD80 (cat. no. 375409, Biolegend, USA) and CD68 (cat. no. 333810, Biolegend, USA) antibodies after the gating strategies (Fig. S2).

### Isolation and Characterization of sEvs

For each UC-sEv manufacturing run, frozen UCMSCs from the PPCB (P3) were thawed and cultured in an Ev-removed culture system. The basal medium, serum-free replacement medium, and phosphate-buffered saline (PBS) were ultracentrifuged at 160,000 × *g* for 8 h to eliminate vesicle analogues before further use. When the 4th, 5th, and 6th generation cells reached approximately 90% confluence, the supernatant (conditioned culture [CM]) of the UCMSCs was collected and stored at − 80 °C. The CM from a cell viability rate of > 90% was used for the subsequent extraction of UC-sEvs. We collected approximately 20 L of CM from P4-P6 generation cells and concentrated it 25-fold via tangential flow filtration (TFF) using a SpectrumLabs KrosFlo Pilot Plus system (USA) equipped with a 300 kDa hollow fiber column (Acefiber, China). The samples were subsequently centrifuged at 3,000 × *g* for 20 min to remove cellular debris, followed by centrifugation at 16,500 × *g* (Beckman Coulter Optima XPN-100 ultracentrifuge, Beckman Coulter, Brea, CA, USA) for 30 min at 4 °C and at 120,000 × *g* (Beckman Coulter Optima XPN-100 ultracentrifuge, Beckman Coulter, USA) for 1 h at 4 °C to sediment the UC-sEVs. UC-sEvs were then washed in PBS and ultracentrifuged again at 120,000 × *g* for another 1 h. The obtained UC-sEvs were resuspended in PBS and stored at − 80 °C until further use. To assess sEv purity, 1 × 10^9^ particles/mL UC-sEvs were incubated with 5% Triton X-100 (Sigma‒Aldrich, USA) at room temperature for 30 min and analysed via a flow NanoAnalyzer (nFCM, U30E, China).

### Transmission Electron Microscopy

sEv morphologies were visualized through transmission electron microscopy (TEM) as described previously [19]. First, sEv preparations were deposited on 200-mesh Formvar-carbon-coated electron microscopy nickel grids and allowed to adsorb for 10min at room temperature. The samples were subsequently fixed and embedded into the grids. After the grids were dried, they were examined under a transmission electron microscope (HT7800, Hitachi, Japan).

### Cellular Uptake of UC-sEvs

To study the cellular uptake of UC-sEvs, UC-sEvs were first prestained with 5 µL of 10 µM DiD-labelling dye (Yeasen, China) in 1 mL of serum-free medium, incubated for 30 min at 37 °C, and ultracentrifuged at 120,000 × g for 70 min at 4 °C. Next, DiD-sEvs were added to HSF cells or HUVECs seeded into a confocal plate. FITC-phalloidin (Yeasen, China) and DAPI (Thermo Fisher, USA) were used to stain the cytoskeleton and nuclei, respectively. The stained cells were observed under a confocal laser microscope (Leica, SP8, Germany).

### EdU Assay

EdU (5-ethynyl-2’-deoxyuridine) staining was performed via the Click-iT™ EdU imaging kit (Invitrogen, Carlsbad, CA, USA) following the manufacturer’s protocol [20]. UC-sEvs (5 × 10^9^ particles) and EdU (10 µM) were added simultaneously to the cultured MLE-12 cells, and the mixture was incubated at 37°C for 24 h, fixed, and stained with DAPI (Thermo Fisher, USA). The EdU + cells were analysed under a fluorescence microscope (Olympus, Japan). Three replicates were established per group.

### Intracellular Reactive Oxygen Species Generation

Briefly, UVB-induced cell models were cultured for 12 h with UC-sEvs (final concentration: 5 × 10^9^ particles/mL) and detected via a DCFH-DA kit (Beyotime, China). PBS was used as a control. ROS production was measured via a flow cytometer (BD FACSCelesta, USA).

### Animal Experiments

The Institutional Animal Care and Use Committee of Northwest University approved all the animal experiments (approval number: NWU-AWC-20211201 M, 4 December 2021), which were performed under the ARRIVE (Animal Research: Reporting of In Vivo Experiments) guidelines. BALB/c mice (male; age: 7–8 weeks; *n* = 120; Beijing Charles River Laboratory Animal Technology Co., Ltd.) were housed in a standard animal facility under controlled conditions (12-h light/dark cycle, temperature of 22–24 °C and 40–60% humidity) with *ad libitum* access to food and water during the experiments. Humane endpoints were predefined to minimize animal suffering. Mice exhibiting signs of distress, severe illness or immobility were humanely euthanized via an overdose of pentobarbital sodium (30 mg/kg, intraperitoneal injection).

### Mouse Groups and Therapeutic Protocols

In total, 48 mice (*n* = 16/group) were intratracheally injected (IT) with 8 U/kg bleomycin (BLM, Solarbio, China) for mortality testing. The mice in the other group (*n* = 48) used for therapeutic effect testing were intratracheally injected with 6 U/kg BLM. All the mice were grouped according to their body weights on the third day before therapy. During the treatment, 12 mice were placed in a transparent box with one hole. The box was connected to the atomization device. Each atomization step lasted for approximately 20 min. The UC-sEv group was nebulized with 4 × 10^10^ UC-sEv particles suspended in 4 mL of PBS twice a day after BLM exposure. The other groups, including the control group (NC + PBS) and model group (BLM + PBS), received equal volumes of PBS. After 5 days of treatment (D7), samples from the inflammation observation group were collected (D8), whereas those from the fibrosis observation group were nebulized for a total of 10 days (D12). The bronchoalveolar lavage fluid (BALF) and lungs of the mice were harvested (D26) for subsequent analysis (Fig. 3B). Because BALF may affect lung morphological analysis and compromise lung function, BALF was collected from half of the mice (*n* = 6) in each group, and the remaining half were used for collecting lung tissue for subsequent analysis. Healthy mice were used as the negative control (NC + PBS group).

### Lung Wet/dry Weight Ratio

To compare the lung water content, the mice were euthanized, and the weight of the wet lung tissue was determined through weighing. The tissues were subsequently dehydrated for 24 h in a drying oven at 65 °C and weighed again to obtain the dry weight. The lung wet/dry weight ratio was also determined.

### BALF Collection and Cell Counts

First, a polyethylene cannula was inserted into the trachea. BALF was collected through the cannula by carefully and slowly instilling and withdrawing PBS (1 mL) into the lung. In total, 3.0 mL of PBS was collected (three times) from each mouse. The BALF was centrifuged at 300 × *g* for 10 min at 4 °C. The cells in the BALF (*n* = 6/group) were pelleted to determine differential cell counts and total cell counts via a five-classification blood cell analyser for animals (DF55 Vet, DYMIND, China) or via a fluorescence cell analyser (CountStar Rigel S2, Ruiyu Biotech Co., Ltd., China). The protein concentration in the BALF supernatant was determined through a bicinchoninic acid assay (Beyotime, China).

### Nanoflow Cytometry Analysis

nFCM (U30E, China) was used to analyse the particle concentration, size distribution, and immunofluorescence staining as described previously [21]. Briefly, the side scatter light (an FF01-488/10 bandpass filter for a 488-nm laser) and fluorescence light (an FF01-525/40 bandpass filter for green fluorescence and an FF02-670/30 bandpass filter for red fluorescence) of individual UC-sEvs were detected simultaneously.

### Immunofluorescence Staining

Immunofluorescence staining for nFCM analysis was performed as described previously [21]. APC-conjugated anti-human CD81 (B384808, Biolegend, USA) and FITC-conjugated anti-human CD9 (312104, Biolegend, USA) were first added to two separate UC-sEv preparations of 20 µL each (particle concentration: ~1 × 10^10^ particles/mL), mixed, incubated at 37 °C for 30 min, and then subjected to nFCM analysis.

To perform confocal laser scanning microscopy as previously described [19], frozen sections of lung tissues or adherent cells were first fixed on slides. The slides were incubated with primary antibodies against rat anti-mouse CD68 (ab53444, Abcam, UK), rabbit anti-mouse α-smooth muscle actin (α-SMA) (19245, CST, USA), and rat anti-mouse CD31 (ab7388, Abcam, UK). The sections were then stained with the secondary antibodies goat anti-rat IgG HL (Alexa Fluor 488, ab150157, Abcam, UK) or goat anti-rabbit IgG HL (Alexa Fluor 594, ab150080, Abcam, UK). The nuclei were stained with DAPI (Thermo Fisher, USA). All the stained sections were imaged under a confocal laser microscope (Leica, SP8, Germany).

### Mouse Groups and Therapeutic Protocols

The Institutional Animal Care and Use Committee of Northwest University approved all the animal experiments (approval number: NWU-AWC-20211201 M, 4 December 2021), which were performed under the ARRIVE (Animal Research: Reporting of In Vivo Experiments) guidelines. BALB/c mice (male, age: 7–8 weeks, *n* = 120; Beijing Charles River Laboratory Animal Technology Co., Ltd.) were housed in a standard animal facility during the experiments. Detailed descriptions of the mouse groups and therapeutic protocols are provided in the Supplementary materials and methods.

### Homing Research in Vivo

Isolated UC-sEvs were stained with DiR (10 µM, Maokang Biotechnology Co., Ltd., China) according to the manufacturer’s instructions and centrifuged at 120,000 × *g* for 70 min. The resulting pellet was resuspended in PBS. Then, BLM-induced BALB/c mice or healthy mice were used and sacrificed at 24 h. The frozen lung tissue sections were fixed, and the slides were analysed through immunofluorescence staining, with all stained sections being imaged via a confocal microscope (Leica, SP8, Germany).

### Lung Histology

The right lobes of the lungs were removed and snap frozen in liquid nitrogen for enzyme-linked immunosorbent assay (ELISA) or quantitative real-time (qPCR) analysis. The left lung was separated and fixed in 4% paraformaldehyde solution. Subsequently, 5-mm paraffin sections were stained with hematoxylin and eosin, Masson, or Sirius Red solution and scanned via a Pannoramic DESK system (3DHistech, Budapest, Hungary). ImagePro software (version 4.0, Media Cybernetics, Silver Spring, MD, USA) was used to record the collagen-occupied areas, which were then divided by the total area examined [6, 22].

### Quantitative Real-Time PCR

The total RNA of the cells and tissues was extracted via the FastPure Cell/Tissue Total RNA Isolation Kit (Vazyme, China), whereas that of the Evs was extracted via the RNeasy Mini Kit (Qiagen, UK). The obtained RNA was reverse transcribed to cDNA by using an All-in-one RT SuperMix Perfect for qPCR (Vazyme, China) or the miRcute Plus miRNA First-Strand cDNA Kit (TIANGEN, China) according to the manufacturer’s instructions. Using the Ultra SYBR mixture (CWBIO, China) or the miRcute SYBR mixture (TIANGEN, China), qPCR was performed on a QuantStudio3 PCR detection system (Thermo Fisher Scientific, USA) to detect the mRNA levels of inflammation-related genes. The RNA and microRNA expression levels were normalized to the β-actin and U6 expression levels. The primers (Table S1) used were designed via DNAman software (Lynnon Biosoft, San Ramon, USA).

### Enzyme-linked Immunosorbent Assay

BALF collection and cell counts are shown in the Supplementary materials and methods. Lung tissue or BALF was obtained from BLM-exposed mice and used for cytokine quantification via ELISA. The lung tissues were homogenized in liquid nitrogen, and the supernatant was collected through centrifugation. The supernatant of the lung tissue or BALF was then used for measuring the levels of TNF-α, IL-6, and IL-1β via a mouse tumor necrosis factor (TNF)-α ELISA kit, a mouse IL-6 ELISA kit, and a mouse IL-1β ELISA kit (KE10007, KE10002, and KE10003, respectively, Proteintech, USA). Following the manufacturer’s instructions, the fluorescence was determined at 450 nm by using a fluorescence microplate reader (Thermo, USA).

### Western Blotting

The samples were lysed via the T-PER Tissue Protein Extraction Reagent (Thermo Fisher Scientific, Rockford, IL, USA). The lysed samples were then treated with rabbit anti-human TSG101 (ab125011, Abcam, USA), GM 130 (11308-1-AP, Proteintech, China), and actin (ab115777, Abcam, USA); rabbit anti-mouse α-tubulin (2144 S, CST, USA) and α-SMA (19245 S, CST, USA); and horseradish peroxidase-conjugated goat anti-rabbit IgG (ab205718, Abcam, UK) antibodies. After the signals were detected via a Chemi Doc XRS + system (Bio-Rad, Hercules, CA, USA), the protein bands were quantified and Analysed via ImageJ 1.34 s software. The relative expression of the target proteins was normalized to that of actin or α-tubulin.

### MiRNA Sequencing

UC-sEv preparations were pretreated with RNase A (Sigma Aldrich) at 37 °C for 1 h to degrade unprotected RNA. Total RNA was subsequently extracted from the UC-sEvs via the Qiagen exoRNeasy Maxi Kit (Qiagen, Hilden, Germany) [23]. The RNA quality was verified via an Agilent Bioanalyzer 4200 (Agilent Technologies, Santa Clara, USA). Small RNA sequencing was performed by Shanghai Biochip Co., Ltd. (Shanghai, China) on the Illumina Nova seq platform (Illumina, San Diego, USA). The known miRNAs were identified via the miRBase v22 database (http://www.mirbase.org/). The quantitative UMI counts were standardized using counts per million values. The differentially expressed miRNAs were calculated and filtered at thresholds of |log2 (FC)| ≥ 1 and *P* < 0.05.

### MiRNA Mimic and Inhibitor Transfection

UCMSCs were transfected with 100 nM miRNA-146a-5p mimic or inhibitor (Thermo Scientific, USA) via Lipofectamine RNAiMAX (Invitrogen, USA) at 60–70% confluence and cultured at 37°C in a 5% CO_2_ humidified atmosphere for 72 h. The supernatant was collected for Ev extraction. UC-sEvs overexpressing miR-146a-5p are named sEv-miR146a+, whereas sEvs with low miR-146a-5p expression are named sEv-miR146a-. qPCR was performed to detect the miRNA-146a-5p expression level.

### Establishment of a BLM Toxicity Cell Model and a TGF-β1-induced Fibrotic Cell Model

MLE-12 cells grown to 80% confluence in 6-well plates were treated with 1640 medium containing 10% FBS and 50µg/mL BLM (Solarbio, China) or TGF-β1 (MCE, USA). The cells were subsequently treated with different types of UC-sEvs (1 × 10^9^ particles/mL) or an equal volume of PBS for 24h under BLM-treated conditions or for 48 h under TGF-β1-treated conditions. Inflammatory factors were detected through ELISA. The level of the α-SMA protein was detected via western blotting.

### Statistics

All the data are expressed as the means ± standard errors. Differences in the data were analysed through Student’s paired *t* test by using GraphPad Prism 5 (GraphPad Software Inc., CA, USA). One-way analysis of variance was employed to compare the means of multiple groups. The statistical significance levels were defined as follows: *P < 0.05, **P < 0.01, ***P < 0.001, and ****P < 0.0001.

## Results

### Setup of the GMPgrade Process and QC Strategy on the Basis of Quality by Design

**Fig. 1:**
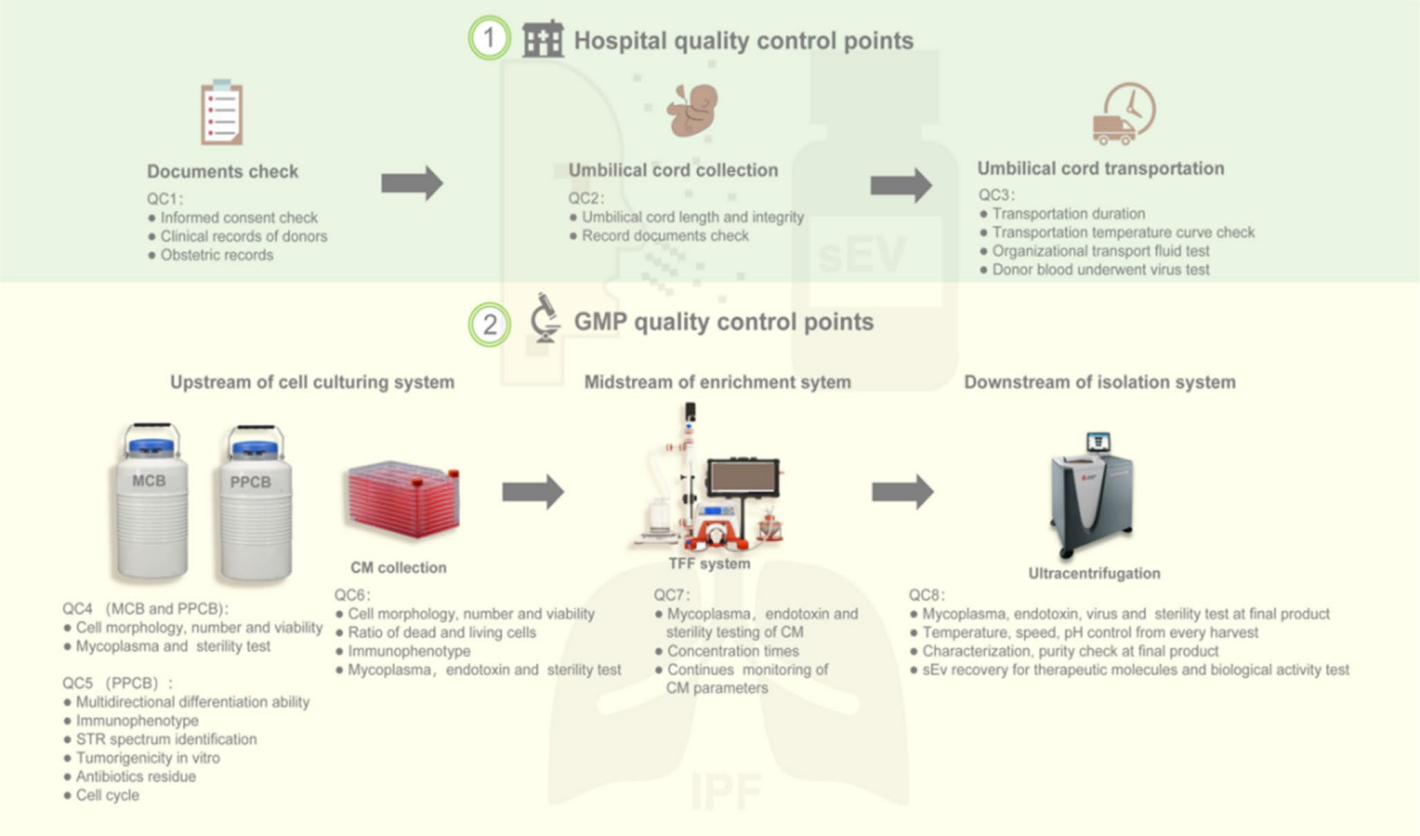
Quality control strategy diagram for the GMP-level UC-sEv. The quality control strategy for GMP-level UC-sEv production includes hospital quality control points and GMP quality control points upstream of the cell culture system, midstream of the enrichment system (TFF system), and downstream of the isolation system (UC system). MCB, master cell bank; PPCB, postproduction cell bank; CM, conditioned medium; TFF, cross-flow filtration; UC, ultracentrifugation

The process of GMP for UC-sEv application was established to ensure that the best practice is followed [18], and a QC strategy for IPF nebulization inhalation (INH) therapy was proposed partly on the basis of the quality by design (QbD) concept [24, 25]. QC measurements were implemented throughout the process of obtaining sEv-containing materials and separating UC-sEvs. We adhered to GMP standards during scale cultivation of UCMSCs and isolation and extraction of UC-sEvs. QC1–QC3 primarily specify the quality control points for umbilical cord collection and transportation (Fig. 1). For umbilical cord collection, written informed consent and ethical approval from a healthy human volunteer were obtained, followed by the acquisition of the donor’s clinical records, which provided information such as patient identity, sex, age, tissue processing, sampling site, collection date, medical history, family genetic history, and anamnesis. Prior to collection, all maternal blood samples from the umbilical cord donors were negative for the virus test, which was conducted by certified laboratories. Supplementary Table S2 presents additional details.

We first comprehensively considered the recommendations of guidelines minimal information for studies of extracellular vesicles (MISEV) 2018 [26], MISEV 2023 [27] released by the International Society for Extracellular Vesicles (ISEV), and guidelines on human MSC-derived sEvs released by the Chinese Research Hospital Association [28] in terms of regulatory standards concerning quality, safety, and efficacy. Accordingly, comprehensive tests were conducted on cell and UC-sEv banks at various stages. Figure 1 presents the QC strategy for GMP-level UC-sEv production, including hospital and GMP quality control points upstream of the cell culture system, midstream of the enrichment system (TFF system), and downstream of the isolation system (ultracentrifugation, UC system). The upstream of the cell culturing system includes two cell banks, a master cell bank (MCB, one generation, P1) and a postproduction cell bank (PPCB, three generations, P3). Furthermore, Ev-depleted medium was used to produce UC-sEvs to achieve a purer product. The use of UCMSCs meeting strict quality control requirements at all cell banks for UC-sEv production ensures that all the UC-sEv-related requirements for clinical use are met [18] in terms of identity, tumorigenesis, functional verification, virus-free content, and sterility (data not shown, Table S2). In this study, the UC-sEv products used were manufactured via a GMP-regulated aseptic operation adopted during production. In accordance with published studies, each patient receiving 1.6 × 10^9^ Ev particles for a single nebulization is safe [29]. Therefore, the safe sterile product (5 × 10^12^ particles) was estimated to meet more than 3000 applications in one operating unit.

### GMP-Grade QC of UC-sEvs Based on the Pathological Mechanism of IPF to Ensure Efficacy in Therapeutic Applications

**Fig. 2:**
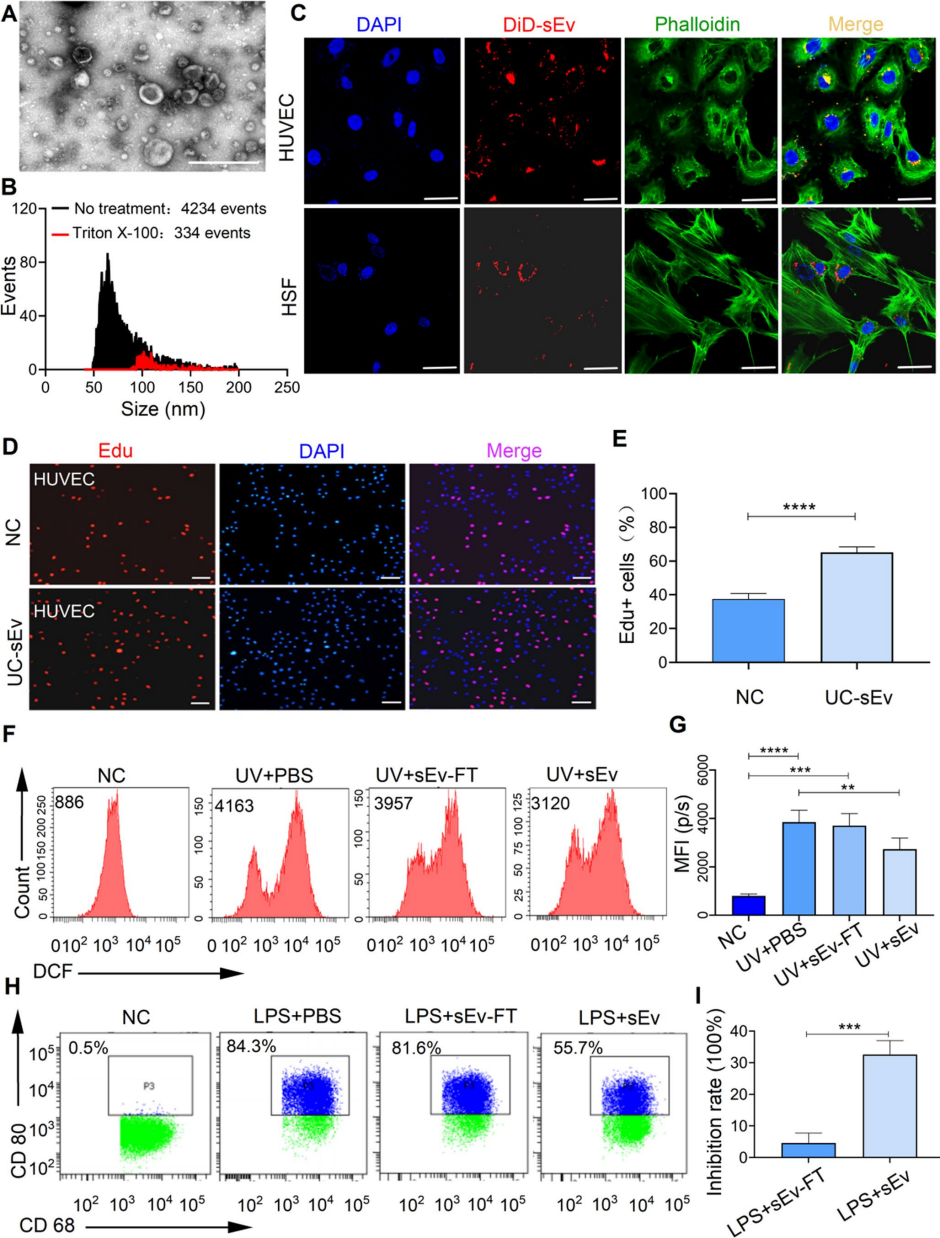
Characterization and functional assays evaluating the therapeutic potential of UC-sEv. **A** Representative TEM micrograph of one UC-sEv isolate batch; scale bar = 500 nm. **B** Size distribution and purity of the particle histograms for a UC-sEv preparation before (black line) and after (red line) Triton X-100 treatment, as determined through nFCM analysis. Triton X-100 treatment was used to estimate sEv purity. **C** Internalization of DiD-labelled UC-sEvs (DiD-sEv) by HUVECs and HSFs. The cytoskeleton was stained with ghost pencyclic peptide (green); scale bar = 100 μm. **D** Representative microscopy images of HUVECs treated with or without UC-sEvs for the EdU assay and **E** quantitative analysis of the data. Scale bar = 50 μm. Reduction in the intracellular ROS level **F** and relative analysis **G** in HUVECs. FT = freeze–thaw. Representative flow cytometry plots of the ability of UC-sEvs to inhibit M1 macrophage polarization **H** and the corresponding inhibition rates **I**. The data are representative of three UC-sEv batches. **P < 0.01, ***P < 0.001, ****P < 0.0001; NC = negative control

A major issue in the GMP of sEvs is establishing an identification method and release standards at various levels of operating systems, including physical, chemical, and bioactivity function characteristics. The stability of this process was verified on the basis of the concentration, characteristics, and purity of the three UC-sEv batches produced (Table S3). The UC-sEvs had a typical round shape, as noted via TEM (Fig. 2A). Most UC-sEv particles ranged in size from approximately 40 mm to 120 nm (< 200 nm) (Fig. S1A), which is consistent with the designation of sEvs according to recent consensus guidelines MISEV 2018 [26]. The average medium or mean particle diameter of the three UC-sEv batches was 60.25–67.75 nm. This size is considered suitable for sEv delivery to the distal lung [30–32]. sEv-specific positive markers (CD81, CD9, and TSG101) further confirmed the identity of sEvs (Table S3 and Fig. S1B, C). The purities of the three UC-sEv batches produced via the standard process were relatively consistent and high, with an average of 5.6 × 10^8^ particles/µg protein, which is considerably higher than the recommended limit of ≥ 1 × 10^8^ particles/µg protein [28]. The average proportion of membranous components was 90.2% (Fig. 2B and Table S3). The product was tested for GM 130, a marker of the Golgi apparatus, and the results were negative, indicating that there was no contamination by the Golgi apparatus (Fig. S1C). The aforementioned results demonstrate that the three batches were stable and had relatively consistent biological and physical properties.

During further functional verification, UC-sEvs were found to be taken up by HSFs and HUVECs in vitro, which is the basis for their biological function (Fig. 2C). Cellular apoptosis and oxidative stress damage are core events during lung epithelial cell senescence. This senescence is thus crucial for the initiation and progression of fibrosis in IPF [33]. We found that UC-sEvs promoted HUVEC proliferation, which is beneficial for re-epithelialization (Fig. 2D, E) and significantly reduced the intracellular reactive oxygen species (ROS) levels (Fig. 2F, G). THP-1, a human leukemia monocytic cell line, is commonly used as a model for estimating the modulation of monocyte and macrophage activities [34]. Macrophages can primarily have proinflammatory (M1-like) or anti-inflammatory (M2-like) attributes. The regulatory effect of Evs on macrophages may play a pivotal role in IPF. Notably, UC-sEvs inhibited M1 macrophages (Fig. 2H, I and Fig. S2A, B), but no significant change was observed in the regulation of M2 macrophages by UC-sEvs in vivo (data not shown). Taken together, these results suggest that UC-sEvs retain both regenerative and immunoregulatory functions after large-scale separation, indicating their therapeutic efficacy.

### UC-sEv is Taken Up by Pulmonary Macrophages and Reduces Mortality and the Inflammatory Response in Mice After Bleomycin Injury

**Fig. 3:**
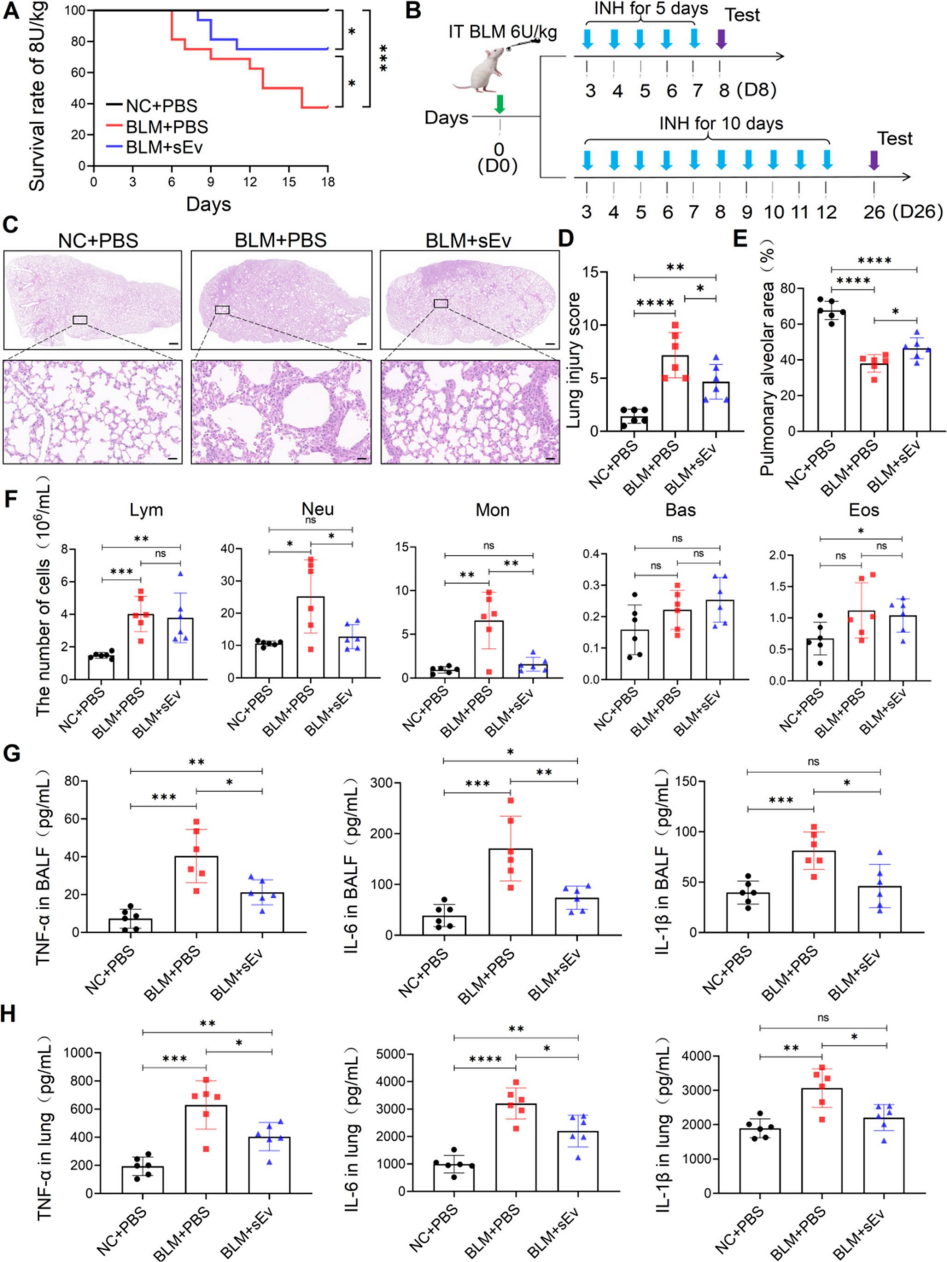
Therapeutic potential of UC-sEv inhalation treatment in IPF mice. **A** Survival of mice treated with or without UC-sEvs throughout the experiment; *n* = 16 per group. **B** Schematics of the UC-sEv study in IPF mice; *n* = 24 per group. IT = intratracheal instillation; INH = inhalation. **C** Representative images of HE-stained lung sections. Top: Scale bar = 100 μm. Bottom: Scale bar = 50 μm. Quantification of the lung injury score **D** and percentage of the pulmonary alveolar area calculated from the HE-stained slices **E**. **F** Counts of lymphocytes (Lym), neutrophils (Neu), monocytes (Mon), eosinophils (Eos), and basophils (Bas), as determined via a small animal blood cell analyser. Detection of TNF-α, IL-6, and IL-1β in BALF **G** and lung tissues **H** through ELISA. The data are presented as the means ± SDs. *P < 0.05, **P < 0.01, ***P < 0.001, and ****P < 0.0001; ns = not significant; NC = negative control

Aerosol nebulization is an effective strategy for transmitting UC-sEvs to the lungs. Our previous studies revealed that UC-sEvs predominantly remain in the lungs after inhalation, and very few or no UC-sEvs are inhaled and directed toward nontargeted tissues (data not shown). To assess the efficacy of inhalation UC-sEvs as a therapeutic intervention for IPF, we used a bleomycin (BLM)-induced mouse model. BLM can induce the collapse of normal lung architecture and attenuate lung function [35]. In accordance with our previous experience, the mice died when exposed to 8 U/kg BLM through the IT. A histological examination revealed direct gross hemorrhagic necrosis (Fig. S3). Therefore, we first investigated whether UC-sEvs, as determined by biofunctionality, can reduce mortality in mice. Detailed weight-related information of the mice in each group was recorded for 26 days (Fig. S4A), and the survival rate was calculated at 18 days. In total, 10 mice (*n* = 16) died after the first mouse died on D6 (Fig. 3A). In contrast, in the BLM with UC-sEv treatment group, mouse death occurred on D8, with a total of 4 mice dying during the entire observation period. Compared with the BLM + PBS group, the BLM with UC-sEv treatment markedly increased the survival rate of the BLM-exposed mice from 37.5% (6/16) to 75% (12/16).

The high inflammatory phase of the IPF model is known to last for 5 days, followed by inflammation resolution and fibrosis progression for 3 weeks [36]. We investigated the effects of UC-sEvs on both short-term inflammation and long-term fibrotic development at D8 and D26, respectively. The recipient mice were nebulized with aerosols of PBS or UC-sEv twice per day from day 3 until day 12 (Fig. 3B). The BLM-exposed mice had greater lung injury scores (7.17 ± 2.14 in the BLM + PBS group; 4.67 ± 1.63 in the BLM + sEv group) than did the NC + PBS-treated mice (1.17 ± 0.68) (Fig. 3D). The BLM with PBS-treated IPF mice presented clear weight loss (Fig. S4B) and lung tissue lesions, characterized by alveolar septum thickening and massive inflammatory cell infiltration (Fig. 3C). In contrast, these effects were improved with UC-sEv inhalation, along with remodelling of the collapsed alveolar architecture, decreased inflammatory cell infiltration, and increased pulmonary alveolar area (Fig. 3E, *P < 0.05 vs. the BLM + PBS group). Detailed blood cell analysis revealed that total protein (Fig. S4C), total inflammatory cells (Fig. S4D), neutrophils (Neu), and mononuclear (Mon) cells (Fig. 3F) in the BALF were significantly reduced after UC-sEv administration. No difference was noted in the number of lymphocytes (Lym), eosinophils (Eos), or basophils (Bas) in the BALF of the BLM-exposed mice treated with UC-sEvs (Fig. 3F). The protein levels of inflammatory cytokines, including IL-6, IL-1β, and TNF-α, significantly decreased in the BALF (Fig. 3G) and lung tissues (Fig. 3H) after BLM with UC-sEv treatment. However, BLM with PBS treatment failed to significantly affect lung inflammation, including pathological changes (Fig. 3C) and changes in the levels of inflammatory cells and cytokines (Fig. 3F–H).

**Fig. 4:**
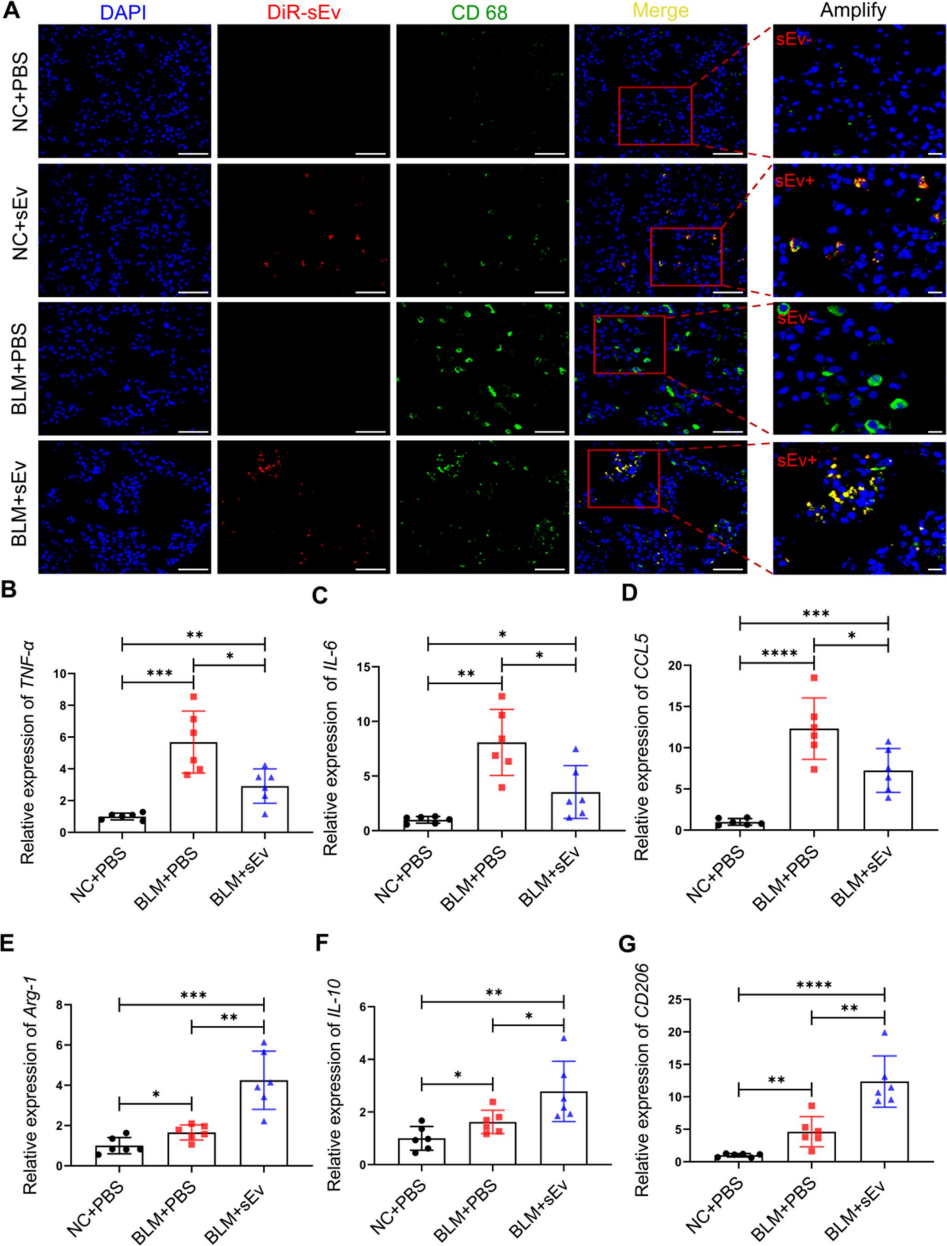
UC-sEvs regulate macrophage function in IPF mice. **A** Representative immunostaining of lung sections for CD68 (green); scale bar = 50 μm. The red signal represents DiR-sEv. Nuclei were stained with DAPI (blue). The red box represents the enlarged area. Relative expression of the M1 macrophage markers *TNF-α* **B**, *IL-6* **C**, and *CCL5* **D** and the M2 macrophage markers *Arg-1* **E**, *IL-10* **F**, and *CD206* **G**; *n* = 6 per group. Each symbol represents 1 mouse. The data are presented as the means ± SDs. *P < 0.05, **P < 0.01, ***P < 0.001, and ****P < 0.0001; NC = negative control

Immunofluorescence staining was performed for CD68, a specific marker of all macrophages [37]. More DiR-labelled UC-sEvs could be taken up by macrophages (CD68+) in diseased mice than in healthy mice, which indicated the homing effect of UC-sEvs after nebulization (Fig. 4A). Therefore, the ability of UC-sEv to regulate the macrophage phenotype in vivo was assessed. M1 macrophages, which are characterized by IL-6, IL-1β, and CCL5 expression, are proinflammatory, whereas M2 macrophages express high levels of anti-inflammatory cytokines (e.g., IL10 and CD206) and exhibit potent arginase-1 (Arg1) activity. Compared with BLM + PBS treatment, BLM + UC-sEv treatment significantly reduced IL-6, TNF-α, and CCL5 mRNA levels (Fig. 4B-D) and significantly increased Arg-1, CD206, and IL-10 mRNA levels (Fig. 4E-G) in lung tissues. In general, UC-sEvs considerably reduced the inflammatory response and enhanced the anti-inflammatory response in BLM-exposed mice. UC-sEv is speculated to be more or less associated with the occurrence of the aforementioned therapeutic effects by regulating macrophage function through homing.

### UC-sEv Effectively Inhibits the Progression of Pulmonary Fibrosis and Protects the Endothelium From Damage in IPF Mice

**Fig. 5:**
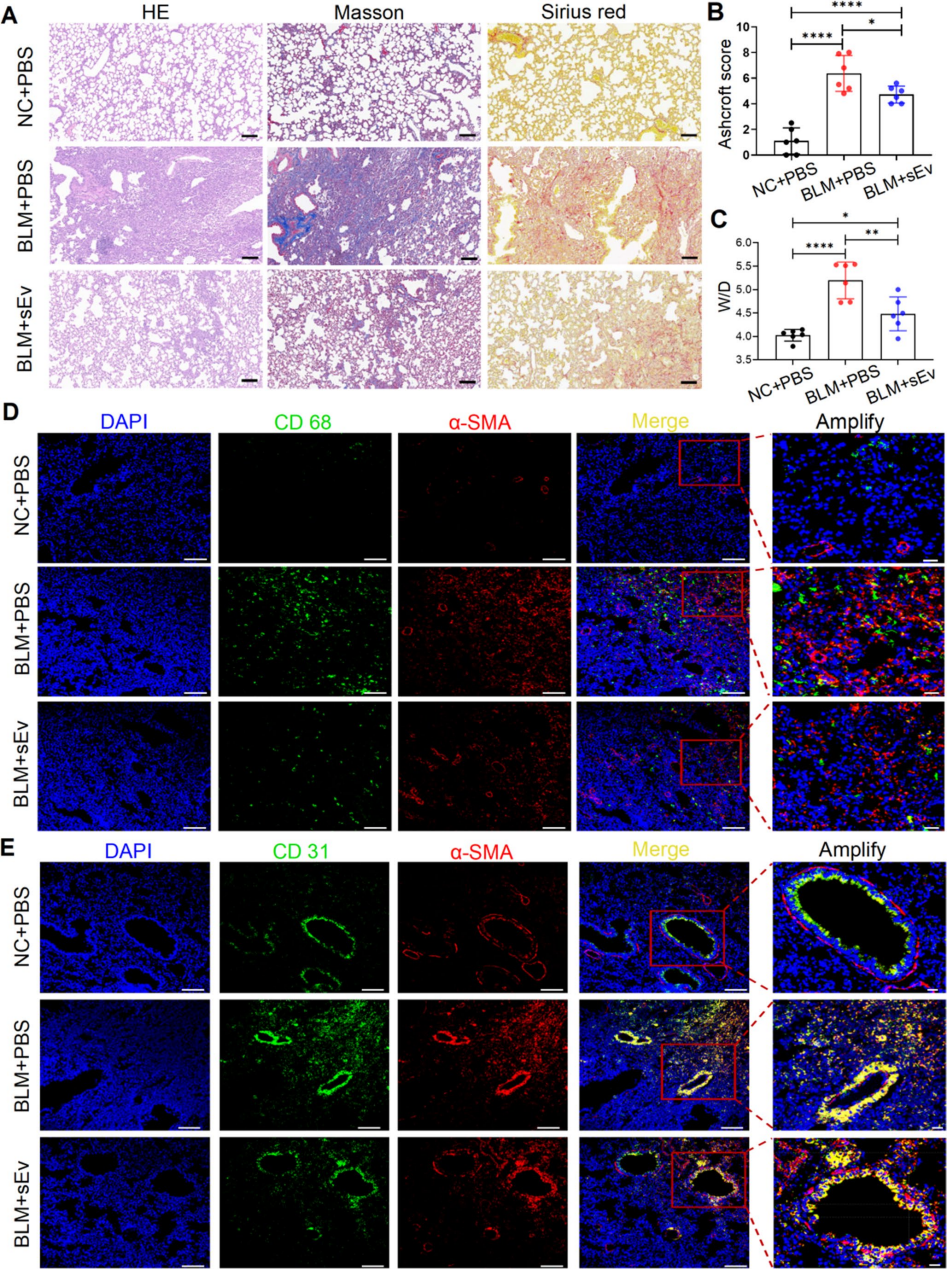
UC-sEvs alleviate fibrosis-related destruction of lung structure and function. **A** Representative images of lung sections stained with HE, Masson’s trichrome, and Sirius red. Scale bar = 100 μm. **B** Lung fibrosis was measured on day 26 on the basis of Ashcroft scores. **C** Comparison of the lung dry/wet weight ratios among the three groups of mice; *n* = 6 per group. The data are presented as the means ± SDs. *n* = 6 per group. Each symbol represents 1 mouse. *P < 0.05, **P < 0.01, and ****P < 0.0001; **D** and **E** Representative immunostaining of lung sections for CD68 (green), CD31 (green), and α-SMA (red); scale bar = 100 μm. Nuclei were stained with DAPI (blue). The red box represents the enlarged area. NC = negative control

BLM-induced pulmonary fibrosis primarily involves collagen deposition that increases tissue density, along with pulmonary edema and enlargement, which directly results in increased lung wet weight [35]. The observed trends in IPF were verified through histopathology. At the pathological level, hematoxylin‒eosin (HE) staining revealed an abnormal alveolar structure and thickening of the alveolar interstitium, along with diffuse pulmonary interstitial fibrosis, in the BLM-treated mice on day 26. In contrast, the alveolar structure was intact and clear in the control group (Fig. 5A). Masson staining and Sirius Red staining revealed that peribronchiolar and perivascular collagen deposition significantly increased in BLM-treated mice, indicating the presence of fibroproliferative disease (Fig. 5A). UC-sEv treatment maintained normal lung morphology even in the presence of BLM toxicity and prevented collagen deposition in the fibrotic lung (Fig. S4E). The decreased Ashcroft score (Fig. 5B) and reduced α-SMA levels in the BLM + UC-sEv group directly support the aforementioned findings and highlight the antifibrotic effect of UC-sEv treatment on α-SMA levels (Fig. S5A, B). The lung wet/dry weight ratio in the BLM + PBS group was significantly greater than that in the NC + PBS group (****P < 0.0001) and BLM with UC-sEv treatment group (**P < 0.01) (Fig. 5C).

Driven by pathological factors, alveolar macrophages, the resident immune cells of the lungs, release inflammatory factors. They also recruit fibroblasts to accumulate in the injured area, ultimately triggering collagen deposition [1]. Interestingly, immunofluorescence staining of the lung tissue revealed that macrophages (CD68+) were considerably more abundant in the α-SMA-positive area in BLM-exposed mice treated with PBS, whereas the number of macrophages and the α-SMA signal decreased in the BLM with UC-sEv treatment group (Fig. 5D, Fig. S5C, D). Compared with the normal lung tissue of the control group, immunofluorescence staining of the fibrotic lung tissue revealed that the number of blood vessels in the α-SMA area increased, suggesting that smooth muscles in the pulmonary vessels of the BLM-exposed mice underwent excessive proliferation (Fig. 5E). Moreover, around the blood vessels/field of the α‐SMA area, the intima (blood vessels/field of the CD31 area) was rough and damaged, contradicting the morphological characteristics of the NC + PBS group. Strong colocalization of CD31 and α‐SMA was noted in the BLM + PBS group. This vascular leakage and fibrosis of surrounding tissues were clearly reduced in the BLM with UC-sEv treatment group. The treatment effect in the BLM with UC-sEv group was greater than that in the BLM with PBS group. In summary, considering the homing effect during the inflammatory phase, UC-sEvs are speculated to regulate macrophage function by transmitting signalling molecules, thereby reducing the induction of pulmonary inflammation, which in turn blocks IPF progression.

### MiRNA Profiles in Three UC-sEv Batches

**Fig. 6:**
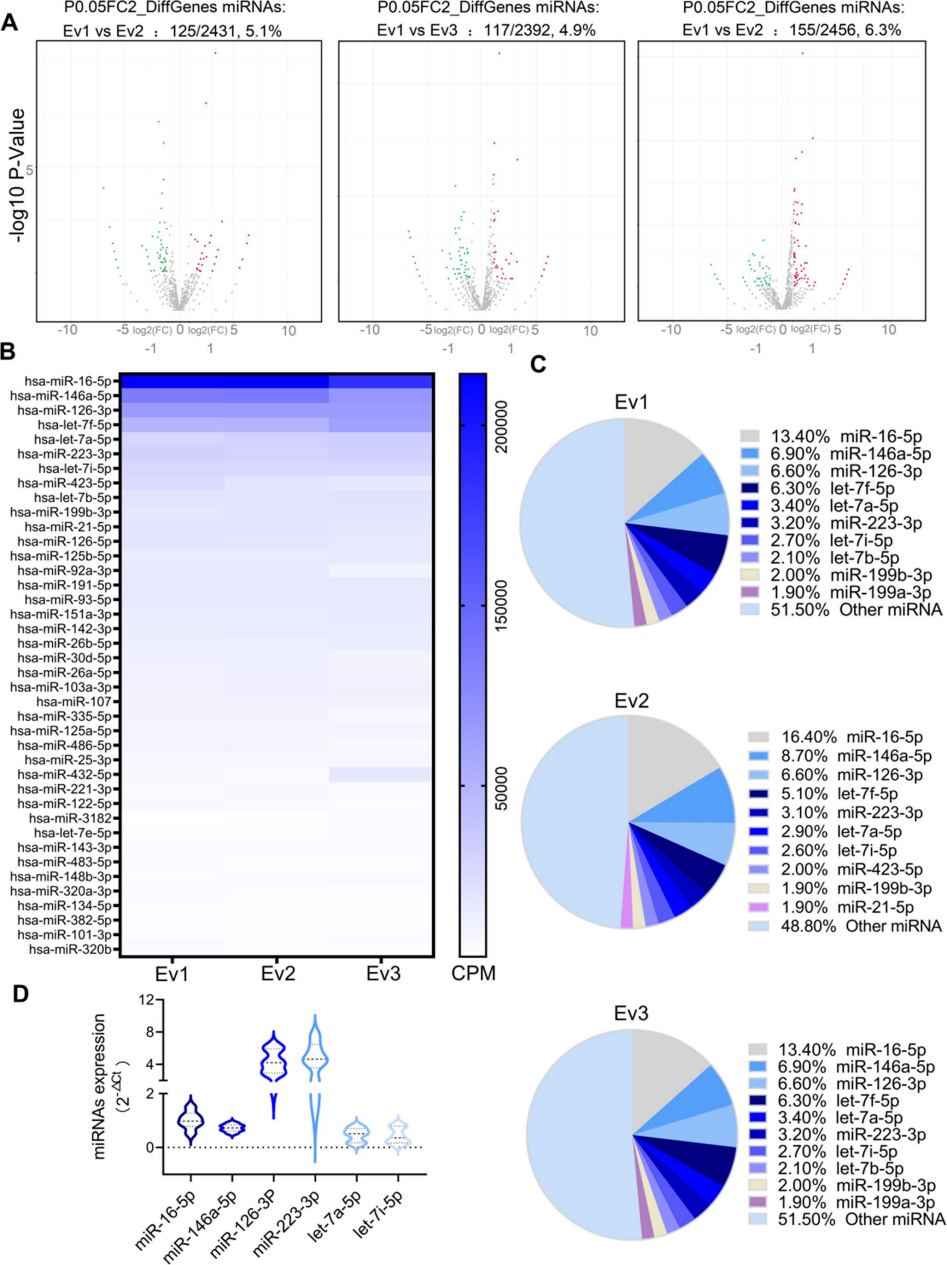
RNA sequencing of three UC-sEV batches. The three UC-sEv batches isolated under GMP were named Ev1, Ev2, and Ev3, respectively, in this experiment. **A** Volcano plot comparing the relative expression of miRNAs in Ev1, Ev2, and Ev3. **B** Heatmap of the most abundant microRNAs in Ev1–Ev3 (top 40). **C** Distribution of the top 10 miRNAs in Ev1–Ev3. More details can be found in Supplementary Table S4. **D** qPCR analysis for verifying the expression profiles of microRNAs expressed in the six batches of UC-sEvs with CV%. More details can be found in Supplementary Table S5

miRNAs in sEvs are crucial cargos for regulating the crosstalk between cells and revealing unique miRNA clusters under specific separation processes. To identify the therapeutic molecules through which UC-sEvs improve IPF, miRNA expression patterns in the three UC-sEv batches were analysed. Overall, over 2300 miRNAs were detected in these samples. Among them, 155 miRNAs (log2-fold change absolute value > 1, *P* < 0.05), approximately 6.3% of the total miRNAs, were differentially expressed between Ev2 and Ev3, indicating that the batches were consistently stable (Fig. 6A). Here, we focused predominantly on miRNAs with high abundance and no differences. The top 40 miRNAs enriched in the three sample batches were slightly different (Fig. 6B). This makes it likely that the quality control goal of screening a functional miRNA with high abundance and small batch differences as the quality standard will be achieved. The proportions of the 10 most abundant miRNAs in Ev1, Ev2, and Ev3 were greater than or close to 50% (Fig. 6C and Table S4), and the 10 most abundant miRNAs in UC-sEvs were selected for further validation. On the basis of the functions of the previously reported top 10 miRNAs, several miRNA molecules involved in UC-sEvs, which might be relevant to our study, were screened out, including let-7a-5p [38], miR-146a-5p [39–42], miR-126-3p [43], miR-16-5p [44], miR-223-3p [45, 46], and let-7i-5p [47]. These miRNA molecules play a positive role in anti-inflammatory and antifibrotic effects. We performed qPCR for the aforementioned miRNAs in six UC-sEv batches (Fig. 6D and Table S5). miR-146a-5p was enriched in UC-sEvs, with an interbatch difference (CV%) of < 20% (Table S5). Furthermore, reduced miR-146a-5p is a biomarker of infant respiratory diseases that contribute to immune dysregulation [48].

### UC-sEv Treatment Alleviated BLM-induced Inflammatory and Fibrosis Responses Through the miR-146a-5p/TRAF6/IRAK1 Axis

**Fig. 7:**
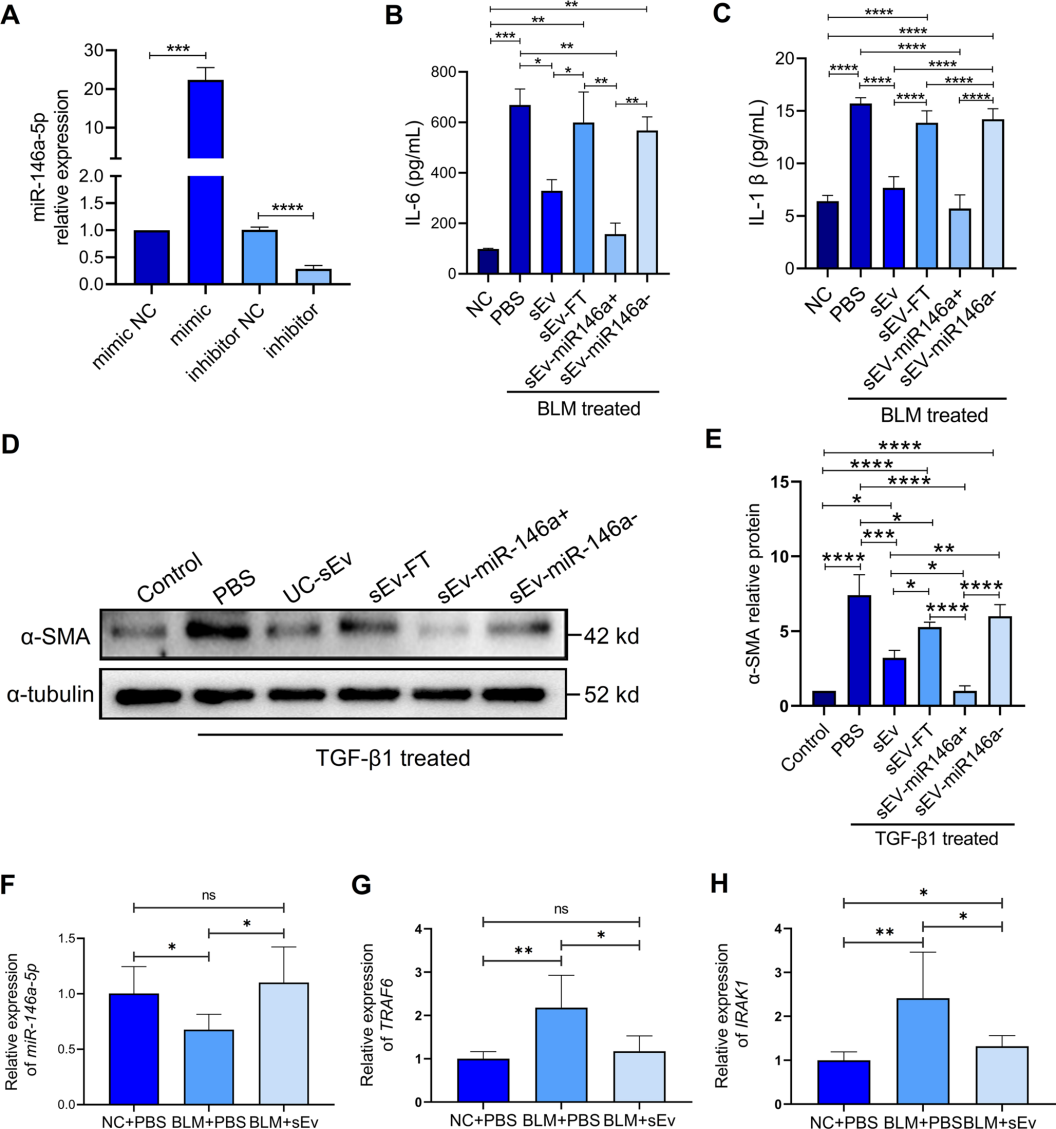
The miR-146a-5p/TRAF6/IRAK1 axis inhibited inflammation and the fibrosis response in cell models and lung tissues. **A** Changes in miR-146a-5p expression in sEvs derived from UCMSCs after a miR-146a-5p mimic or inhibitor was added exogenously. The inflammatory cell model was established by exposing MLE-12 cells to BLM. ELISA was performed for IL-6 **B** and IL-1β **C** after treatment with different sEv types. sEv = UC-sEv; sEv-FT = UC-sEv with repeated freeze–thaw cycles; sEv-miR146a + = UC-sEv with upregulated miR-146a-5p expression; sEv-miR146a- = UC-sEv with downregulated miR-146a-5p expression. **D** Western blot analysis of the protein levels of fibrosis-related factors. α-SMA expression in the TGF-β1-stimulated MLE-12 cells supplemented with different sEv. **E** Statistical analysis of the expression levels of α-SMA normalized to those of α-tubulin. qPCR analysis of miR-146a-5p **F** and the target genes *traf6* **G** and *irak1* **H**. The data are presented as the means ± SDs. *P < 0.05, **P < 0.01, ***P < 0.001, and ****P < 0.0001; ns = not significant; NC = negative control

To study the roles of miR-146a-5p in the inflammatory and fibrosis response, the miR-146a-5p mimic and inhibitor were transfected into UCMSCs. Different UC-sEv types were derived from the transfected UCMSCs. Figure 7A presents the high efficacy of transfection, as examined through qPCR. To further determine whether specific miR-146a-5p decreases inflammatory factor release and inhibits fibrosis in lung epithelial cells, the direct effects of miR-146a-5p on the biology of MLE-12 cells were examined. These cells were treated with UC-sEv (sEv), UC-sEv-TF (sEv-FT), or UC-sEv-miR-146a-5p+/miR-146a-5p- (sEV-miR-146a+/sEV-miR-146a-) after BLM-induced injury. The ELISA results revealed that only UC-sEvs, as well as sEv-miR-146a+, effectively inhibited the release of inflammatory factors, including IL-6 (Fig. 7B) and IL-1β (Fig. 7C), from BLM-treated MLE-12 cells. However, UC-sEvs subjected to repeated freeze–thaw cycles had no ability to inhibit inflammation, which indicated that UC-sEvs can reduce the BLM-induced secretion of inflammatory factors. A significant reduction in anti-inflammatory ability was observed in the samples with miR-146a-5p downregulation (Ev-miR-146a-) compared with the Ev-miR-146a + group (Fig. 7B, **P < 0.01; Fig. 7C, ****P < 0.0001). The ability of miR-146a-5p to inhibit fibrosis was verified in TGF-β1-stimulated MLE-12 cells. Western blotting revealed that sEv (***P < 0.001 vs. the PBS group)- or sEv-miR-146a+ (****P < 0.0001 vs. the PBS group)-induced significant decreases in α-SMA protein expression in the TGF-β1-stimulated MLE-12 cells (Fig. 7D, E), suggesting the antifibrotic effect of miR-146a-5p. miR-146a-5p downregulation had no significant inhibitory effect on fibrosis, whereas miR-146a-5p upregulation was beneficial for inhibiting fibrosis (*P < 0.05 vs. the sEv-miR146a- group).

On the basis of preliminary data, we speculated that UC-sEv regulation during the inflammatory phase of lung injury is key in delaying IPF progression. Therefore, we noted changes in the expression of miR-146a-5p and its target genes in early inflammatory lung tissue samples. miR-146a-5p has a special appeal, as it induces a negative feedback loop. This downregulated the expression of proinflammatory mediators by targeting TNF receptor-associated factor 6 (TRAF6) and interleukin-1 receptor-associated kinase 1 (IRAK1) [49, 50]. The miR-146a-5p expression level was significantly greater after UC-sEv treatment than after PBS treatment (Fig. 7F). Furthermore, TRAF6 (Fig. 7G) and IRAK1 (Fig. 7H) mRNA expression significantly decreased in lung tissues on D8 after UC-sEv treatment. These data suggest that miR-146a-5p plays an indirect role in anti-inflammatory and antifibrotic effects through the negative regulation of the downstream target genes *traf6* and *irak1*. Overall, miR-146a-5p was enriched in UC-sEvs produced under GMP guidance and expressed stably in different batches; therefore, it is suitable as a potent molecule for IPF treatment. This finding further clarifies the quality standards for UC-sEvs used in IPF clinical treatment (Table 1).

**Table 1:** Product quality standards of GMP-grade UC-sEv for IPF

Parameter	Method	Release criteria
Security		
Sterility	BacT/ALERT	Negative
Bacterial endotoxin (EU/mL)	According to Ch.P	< 0.5
Mycoplasma	qPCR	Negative
*HIV*,* HCV*,* HBV*,* HTLV*,* EBV*,* HCMV*,* TP*	qPCR	Negative
Physical indicators		
Appearance	Visual	Clear and free of foreign objects
pH	pH meter	[5.5, 7.5]
Osmotic pressure	freezing-point method	280 ~ 310 mmol/L
Characterization		
Morphalogy	TEM	Membrane vesicles with intact morphology and lipid bilayer
Nano parameters	nFCM	Median particle size [50, 80]
	nFCM	Average particle size [50, 80]
Characteristic protein	nFCM	CD 81^−^ and CD 9^−^ ≤ 30%
	WB	TSG 101 positive
Purity		
Particles/protein	nFCM and BCA	≥ 4 × 10^8^ particles/µg protein
Negative protein	WB	GM 130 negative
Proportion of membrane components	Triton X-100	≥ 80%
Therapeutic molecule		
miRNAs	qPCR	miR-146a-5p positive
Biological activity		
Uptake by cells	Confocal imaging	Uptake by HSF or HUVEC
HSF Proliferation	EdU assay	Promote cell proliferation
ROS level	Flow cytometry	Inhibit ROS generation
Immunomodulatory function	Macrophage polarization assay	Inhibit M1 macrophage polarization

*Ch.p.* Chinese Pharmaceutical, *nFCM* flow NanoAnalyzer, *TEM* transmission electron microscopy, *ROS* reactive oxygen species

## Discussion

Given that the action of MSCs can be predominantly paracrine, MSC-Ev therapies are speculated to be more advantageous than MSC-based therapies. Before MSC-Evs are truly applied in clinics, a key aspect is the isolation of sEvs through a validated, scalable, and GMP-compliant production process. This would allow products that meet certain quality standards suitable for clinical applications of MSC-Evs against specific diseases to be obtained. Our study focuses predominantly on the quality control strategy of GMP-grade UC-sEvs based on QbD and investigates the mechanisms underlying the therapeutic action of UC-sEvs to support the formulation of quality standards ensuring the effectiveness of clinical treatment for IPF. Under the guidance of quality control strategies and by investigating GMP-grade quality control, pharmacological mechanisms, and key miRNA components, for the first time, we provide a quality control standard for the clinical treatment of IPF through nebulization inhalation of UC-sEvs (see the Graphic abstract).

To become a true drug for lung regeneration, a GMP-grade UC-sEv must be purified after production, followed by its characterization and identification, including its physical configuration and bioactivity function characteristics. Here, we established the GMP process on the basis of previous experience [18] and a quality control strategy for producing UC-sEvs for clinical applications. On the one hand, we achieved large-scale production of GMP-guided UC-sEvs, and each operating unit can meet the treatment needs of over 3000 times. The TFF system with a 300 kDa cut-off membrane, which was employed for medium harvest, was beneficial for downstream purification because the volume of the harvested CM was reduced. On the other hand, an identification method, including identification, characterization, and bioactivity characterization, was established, which provides control strategy considerations for IPF clinical treatment via the use of UC-sEvs. Our quality control strategy involves the following distinguishing features: (i) the establishment of functional research indicators on the basis of IPF pathological characteristics; (ii) the validation of therapeutic effects in IPF animals following clinical administration; and (iii) the identification of key therapeutic molecules with guaranteed clinical efficacy as quality control standards. Another important aspect in sEv therapeutic development is identifying and quantifying the key features determining their identity, purity, sterility, potency, and stability, thereby ensuring batch-to-batch reproducibility of their therapeutic efficacy. Supplementary Table S3 presents a detailed record of three GMP-grade UC-sEv batches, whose main characteristics, such as identity, purity, sterility, and potency, were not significantly different. The process stability ensures the therapeutic efficacy of the product.

Considering that immune cell dysfunction [51] and oxidative stress [52] play crucial roles in IPF progression, an IPF treatment mechanism-based quality control strategy was applied to release standards of the products. When the three UC-sEv batches were functionally identified, we noted that UC-sEv plays a substantial role in endothelial protection and immune regulation by promoting endothelial cell proliferation, decreasing ROS generation, and inhibiting M1 macrophage proliferation. The aforementioned settings are beneficial for ensuring product quality and eliminating process deviation-induced batch instability. However, analysing the active ingredients of a biologically active therapeutic drug is crucial. The lack of clarity on this issue may lead to unstable therapeutic effects resulting from interbatch differences. Our study focused on deciphering the key efficacy molecules of IPF clinical treatment and developing sEv quality standards suitable for this treatment. In the subsequent step, to screen for effective molecules, the miRNAs of UC-sEvs were sequenced. We also explored the efficacy of UC-sEvs against IPF and the underlying mechanism.

Next, we assessed the efficacy of UC-sEv treatment in a preclinical IPF model by using one batch of verified UC-sEvs. UC-sEv inhibited lung injury-induced mouse death and resulted in a higher mouse survival rate than did BLM + PBS. The therapeutic effects of UC-sEv and PBS on alleviating inflammation and inhibiting fibrosis were compared in IPF mice treated with 6 U/kg BLM. These data suggest that early UC-sEv intervention through nebulization can improve the inflammatory response of IPF mice, as evaluated through three examinations, namely, histology, BALF, and lung tissue. TNF-α, IL-6, and IL-1β are inflammatory cytokines that play crucial pathogenic roles in the initial phase of lung injury and inflammation [53, 54]. The expression levels of these inflammatory cytokines were significantly reduced after UC-sEv treatment. Our findings indicate that UC-sEv enhances the pulmonary microenvironment of IPF mice by inhibiting the induction of inflammation.

In the early inflammatory response of IPF, various inflammatory cells are involved, which can directly or indirectly lead to IPF, at least partially by regulating the lung macrophage phenotype [55], thereby playing a core regulatory role in IPF progression [56]. Homing differences in DiR-sEv were noted between IPF and healthy mice. The CD68 + cells of IPF mice strongly colocalized with UC-sEvs. These findings indicated that macrophages can engulf nebulized UC-sEvs in IPF mice. More intuitively, immunofluorescence revealed that these macrophages tend to aggregate in clusters. Considering that the distribution characteristics of macrophages at the site of injury differ from those in normal tissues, we believe that macrophages tend to aggregate in diseased tissues. Whether this uptake further affects macrophage function has been further demonstrated by decreases in the levels of M1 macrophage markers such as IL-6, TNF-α, and CCL5 and increases in the levels of M2 macrophage markers such as Arg-1, CD206, and IL-10. Overall, according to our observations, UC-sEv administration effectively blocked BLM-induced inflammation, at least partially because of the regulation of macrophage function. This result may directly affect the fibrosis process in IPF mice. In the fibrotic lung, changes in macrophages were noted before and after treatment. Our data suggested that in IPF mice, the strongly fibrotic region was accompanied by numerous macrophages, whereas in BLM with UC-sEv-treated mice, the fibrotic signal was weakened, and the number of macrophages was significantly reduced. In addition to the inflammatory pathway, the epithelial pathway, as another main signalling pathway, together dominates fibrogenesis in the lung [35, 57]. Interestingly, through HE analysis of short-term inflammatory tissue, we found that BLM increased total cell counts and total protein concentrations in the BALF, suggesting damage to the peritracheal blood vessels in the model mice. As fibrosis gradually worsens, microvascular density also progressively decreases, whereas vascular permeability increases. In the area of fibroblast aggregation, capillaries are almost absent. According to our results, in areas with severe fibrosis, blood vessels are severely damaged and exhibit associated structural damage to the intima. UC-sEvs to some extent protect the intima from damage and maintain a relatively clear morphological structure.

In theory, a stable production process can ensure the stability of the composition of MSC-specific miRNAs with relatively high expression. High levels of MSC-specific miRNA transmission may be implicated in anti-inflammatory and antifibrotic effects. On the basis of our standardized and scaled production and quality control, we will have sufficient evidence to validate this hypothesis to search for highly enriched and batch-stable miRNAs for IPF therapy. RNA-seq of three UC-sEv batches was completed, and a miRNA cluster in those products was identified from more than 2300 miRNAs. Among them, the top 10 enriched miRNAs accounted for approximately 50% of the respective sample analyses. The aforementioned data indicate that through standardized production, the consistency of sEvs can be ensured in terms of their characteristics, purity, and biological functions and their expression of miRNA profiles. qPCR analysis revealed that miR-146a-5p, a miRNA with anti-inflammatory properties [58], was enriched in UC-sEvs, with an interbatch difference of CV% < 20%. Therefore, we further postulated that UC-sEv-derived miR-146a-5p is crucial for ameliorating the effects of BLM toxicity.

To further demonstrate that the therapeutic effect of UC-sEvs is conferred by the active substance they carry, UC-sEvs repeatedly freeze-thawed were used as a negative control for subsequent studies. To maintain consistency with animal models, MLE-12 cells were also exposed to BLM to promote high expression of inflammatory factors. BLM-exposed MLE-12 cells expressed high levels of inflammatory factors, and the addition of UC-sEvs, rather than repeatedly freeze-thawed sEvs (sEv-FTs), inhibited IL-6 and IL-1β protein expression. However, when miR-146a-5p was inhibited, the previously observed inflammatory inhibitory ability disappeared. Furthermore, UC-sEv-delivered miR-146a-5p attenuated fibroblast activation through the inhibition of α-SMA expression. Compared with sEv- or sEv-miR146a + MLE-12 cells, sEv-miR146a-treated MLE-12 cells caused no inhibition of α-SMA expression. miR-146a-5p tightly regulates inflammation strength by targeting signalling proteins involved in innate immune responses, such as TRAF-6 and IRAK-1 [59]. The expression of miR-146a-5p and its target genes was further validated in the lung tissue of IPF mice. The miR-146a-5p expression level was inhibited in IPF mice, whereas UC-sEv treatment increased this expression level and decreased the expression of the target genes *traf6* and *irak1*. Our data suggested that miR-146a-5p expression levels markedly decreased after BLM injury and that their expression was augmented in lung tissue or cells after UC-sEv treatment. This treatment resists inflammation and fibrosis by regulating *traf6* and *irak1* in lung tissue.

Finally, on the basis of the in vitro function, animal efficacy, and identification of key therapeutic molecules, we propose quality control standards for UC-sEv nebulization therapy for IPF (Table 1). The standard involves a more comprehensive, more specific characterization of the main therapeutic attributes of UC-sEvs. Specifically, the quality standards for certain parameters were expanded or improved to ensure product effectiveness. For example, determining the particle-to-protein ratio is a common method of assessing the purity of Ev samples [26, 28], and the proportion of membrane components was added as a quality standard for purity. With respect to improvement in quality standards, the particle-to-protein ratio of the three UC-sEv batches was approximately 5× 10^8^ particles/µg protein, which is considerably higher than that stated in the guidelines of the Chinese Research Hospital Association (1 × 10^8^ particles/µg protein). Therefore, we have increased this standard to 4 × 10^8^ particles/µg protein for quality control.

The current study has several limitations that should be addressed. We found that UC-sEv may affect macrophage function during the short-term inflammatory phase, thereby inhibiting the hyperfibrotic response. However, direct evidence that sEvs affect IPF occurrence and development by regulating macrophage function both in vivo and in vitro is lacking. On the other hand, direct evidence regarding the involvement of miR-146a-5p in IPF treatment is lacking. A comparison of the therapeutic differences between the miR-146a-5p-overexpressing and miR-146a-5p-knockdown sEv groups will be meaningful for attaining a deeper understanding of the mechanism of action of sEvs in IPF treatment. The main aim of this study was to identify the therapeutic effects of GMP-grade UC-sEvs under a quality control strategy and to develop corresponding quality standards for UC-sEv treatment of IPF, thereby promoting the development of clinical research. Therefore, we focused more on the generation and implementation of a standardized application.

## Conclusion

Overall, in this study, a large-scale UC-sEv production process was established in a GMP-compliant cell factory, with each operating unit capable of catering to the treatment needs of more than 3000 times. To the best of our knowledge, we are the first to use quality-controlled UC-sEvs and determine their therapeutic effect and mechanism against IPF. Inhalation of UC-sEvs is an effective approach for IPF treatment and has strong therapeutic effects, as evidenced by the improved survival rate. Specifically, it reduced inflammation and the incidence of pulmonary fibrosis in IPF mice, at least in part by regulating macrophage function. RNA-seq revealed that UC-sEv produced through a standardized method had a stable and consistent MSC-specific miRNA expression profile, and miR-146a-5p, a candidate therapeutic molecule, was selected because of its stability under GMP-grade quality control production. We demonstrated that miR-146a-5p delivered through UC-sEvs produced anti-inflammatory and antifibrotic effects in two cell models and that it negatively regulated inflammation in IPF mouse tissues by targeting TRAF6/IRAK1. Finally, for the first time, we systematically propose quality standards for treating IPF through UC-sEv inhalation on the basis of validation data (Table 1). The quality standard and experimental data provide a robust scientific basis for initiating clinical trials on the efficacy of UC-sEvs in IPF treatment and offer new insights into the mechanisms underlying UC-sEv-mediated amelioration of IPF.

## Supplementary Information

Below is the link to the electronic supplementary material.

ESM 1 (DOCX 2.85 MB)

ESM 2 (DOCX 3.02 MB)

## Data Availability

The data are available in the article and obtained from the corresponding author upon reasonable request. All related miRNA data that support the findings of this study have been deposited in the Sequence Read Archive (SRA) with the accession number PRJNA1214543.

## Abbreviations

sEvs: Small extracellular vesicles
UCMSCs: Umbilical cord mesenchymal stem cells
UC-sEvs: sEvs derived from UCMSCs
QbD: Quality by Design
GMP: Good manufacturing practice
QC: Quality control
PPCB: Postproduction cell bank
MCB: Master cell bank
*HCV*: *Hepatitis C virus*
HBV: *Hepatitis B virus*
HIV: *Human immunodeficiency virus*
TP: *Treponema pallidum*
HCMV: *Human Cytomegalovirus*
HTLV: *Human T-lymphotropic virus*
EBV: *Epstein–Barr virus*
Ch. P: The Chinese pharmacopoeia
TFF: Tangential flow filtration
