# Supplementary material for: Investigating the Therapeutic Efficacy of Quality-Controlled, miR-146a-5p-Enriched Small Extracellular Vesicles Derived From MSCs Against Idiopathic Pulmonary Fibrosis
Source: Stem Cell Rev Rep. 2025 Sep 24;22(1):523–44. doi: 10.1007/s12015-025-10976-8 (PMC12795917; doi:10.1007/s12015-025-10976-8)
Supplement: Supplementary file 1 — (DOCX 2.85 MB) [file 12015_2025_10976_MOESM1_ESM.docx]

**Supplementary File 1**

**Investigating the therapeutic efficacy of quality-controlled, miR-146a-5p-enriched small extracellular vesicles derived from MSCs against idiopathic pulmonary fibrosis**

Xin Wang ^1,*^ , Lingjiao Meng ^1,*^ , Qiuhong Wang^1^, Ruixue Rong^1^, Yu Zhang^3^, Xiaohui Zhao^1^, Chen Liang^1^, Huizhen Guo^1^, Li Deng^4^ , Zengqi Tan^5^, Feng Guan^6^ , Yi Tan ^1,2^

^1^Qilu Cell Therapy Technology Co., Ltd, Gangyuan 6th Road, Licheng District, Ji'nan, Shandong 250000, P. R. China.

^2^Shandong Yinfeng Life Science Research Institute, Ji'nan, P. R. China.

^3^Department of Otolaryngology, Head and Neck Surgery, Yantai Yuhuangding Hospital, Qingdao University, Yantai, P. R. China.

^4^Department of Gastroenterology, The First Affiliated Hospital of Shandong First Medical University & Shandong Provincial Qianfoshan Hospital, Ji'nan, P. R. China.

^5^School of Medicine, Northwest University, Xi'an, P. R. China.

^6^Key Laboratory of Resource Biology and Biotechnology in Western China, Ministry of Education, Provincial Key Laboratory of Biotechnology, College of Life Sciences, Northwest University, Xi'an, P. R. China.

* These authors contributed equally to this work.

**Corresponding author:** Yi Tan

**Address:** Qilu Cell Therapy Technology Co., Ltd., Gangyuan 6th Road, Licheng District, Jinan, Shandong 250000, P. R. China.

**Tel:** +86-0531-88233100 **E-mail:** pkuty@126.com

**Supplementary Tables**

**Table S1. Primers used for qPCR**

| Name | Forward primer | Reverse primer |
| --- | --- | --- |
| m-IL-6 | TAGTCCTTCCTACCCCAATTTCC | TTGGTCCTTAGCCACTCCTTC |
| m-TNF-α | CCCTCACACTCAGATCATCTTCT | GCTACGACGTGGGCTACAG |
| m-CCL5 | ACACCACTCCCTGCTGCTTTG | TCTCTGGGTTGGCACACACTTG |
| m-IL-10 | GCTCTTACTGACTGGCATGAG | CGCAGCTCTAGGAGCATGTG |
| m-Arg-1 | CTCCAAGCCAAAGTCCTTAGAG | AGGAGCTGTCATTAGGGACATC |
| m-CD206 | CTCTGTTCAGCTATTGGACGC | CGGAATTTCTGGGATTCAGCTTC |
| m-TRAF6 | CTCAGCGCTGTGCAAACTATATATCCC | GGCGTATTGTACCCTGGAAGGG |
| m-IRAK1 | TTCCTCCACCAAGCAGTCAAGC | CACCCTCTCCAATCCTGAGTTCTTC |
| m-β-actin | GGCTGTATTCCCCTCCATCG | CCAGTTGGTAACAATGCCATGT |
| m-miR-146a-5p | CCGGCGTGAGAACTGAATTCCATGGGT | Universal reverse primer* |
| hsa-miR-146a-5p | GCACGCGTGAGAACTGAATTCCATGGG |  |
| hsa-miR-16-5p | CGTAGCAGCACGTAAATATTGGCG |  |
| hsa-miR-126-3p | GCCTCGTACCGTGAGTAATAATGCG |  |
| hsa-miR-223-3p | GCTGTCAGTTTGTCAAATACCCCA |  |
| hsa-let-7a-5p | GGCGCCGCTGAGGTAGTAGGTTGT |  |
| hsa-let-7i-5p | GGCTGAGGTAGTAGTTTGTGCTGTT |  |

* Cat No.：CD109 (TIANGEN, China)

**Table S2. QC strategy of GMP-grade UC-sEvs for IPF treatment**

| **QC Point** | **Testing Items** |
| --- | --- |
| QC1 | 1-1 Informed consent check  1-2 Clinical records of donors: identity, gender, age, tissue processing, sampling site, collection date, medical history, family genetic history, and anamnesis  1-3 Obstetric records |
| QC2 | 2-1 Umbilical cord length and integrity  2-2 Record documents check |
| QC3 | 3-1 Transportation duration: ≤ 36 h  3-2 Transportation temperature curve check: 2-8 °C  3-3 Organizational transport fluid testing: microbiology test  3-4 Donor blood underwent bloodborne pathogenic microorganisms: *HCV, HBV，HIV，TP，HCMV，HTLV，EBV* |
| QC4 | 4-1 Cell morphology, number and viability: cell viability ≥ 90%  4-2 Mycoplasma and sterility testing: negative |
| QC5 | 5-1 Multidirectional differentiation ability:osteocytes, adipocytes, and chondrocytes  5-2 Immunophenotype: CD 90, CD 105, CD 73, CD 44 ≥ 95%; CD 45, CD 34, CD 11b, CD HLA-DR ≤ 2%  5-3 STR spectrum identification  5-4 Tumorigenicity in vitro: soft agar cloning; telomerase activity  5-5 Antibiotics residue  5-6 Cell cycle |
| QC6 | 6-1 Cell morphology, number and viability  6-2 Ratio of dead and living cells  6-3 Immunophenotype  6-4 Mycoplasma, endotoxin and sterility testing |
| QC7 | 7-1 Mycoplasma, endotoxin and sterility testing of CM  7-2 Concentration times  7-3 Continues monitoring of CM parameters: pH, osmotic pressure |
| QC8^*^ | 8-1 Mycoplasma, endotoxin, human derived virus and sterility testing at final product  8-2 Temperature, speed, pH control from every harvest  8-3 Characterization, physical indicators, and purity check at final product  8-4 sEv recovery for therapeutic molecules and biological activity test |

QC, quality control; *hepatitis C virus,* HCV; *hepatitis B virus,* HBV; *human immunodeficiency virus*, HIV; *Treponema pallidum,* TP; *human cytomegalovirus,* HCMV; *human T-lymphotropic virus,* HTLV; *Epstein–Barr virus*, EBV; CM, conditioned medium.

* More details are provided in Supplementary Table 6.

**Table S3. Parameter statistics of three UC-sEv batches**

| Batch | Concentration | | | Characterization | | | | | Purity | | | |
| --- | --- | --- | --- | --- | --- | --- | --- | --- | --- | --- | --- | --- |
|  | Particles  (p/mL) | Proteins  (μg/mL) | Total RNA  (μg/mL) | Particle size (Medium/Mean) | CD9^+^ | CD81^+^ | CD9^-^ CD 81^-^ | TSG 101 | sEVs/protein  (p/μg) | sEvs/RNA (p/μg) | Membrane proportion | GM 130 |
| 1 | 3.25E+11 | 596 | 23.23 | 57.25/65.49 | 70.4 | 12.8 | 24.5 | positive | 5.45E+8 | 1.61E+10 | 88.1% | negative |
| 2 | 2.82E+11 | 603 | 23.77 | 60.25/67.62 | 74.4 | 10.9 | 23.8 | positive | 4.68E+8 | 1.79E+10 | 90.5% | negative |
| 3 | 3.80E+11 | 583 | 24.92 | 63.25/70.13 | 67.6 | 11.7 | 28.5 | positive | 6.52E+8 | 1.52E+10 | 92.1% | negative |
| Average | 3.29E+11 | 594 | 23.97 | 60.25/67.75 | 70.8 | 11.8 | 25.6 | - | 5.55E+8 | 1.64E+10 | 90.2% | - |
| CV% | 14.93 | 1.71 | 3.60 | 4.98/3.43 | 4.83 | 8.08 | 9.91 | - | 16.65 | 8.38 | 2.23 | - |

p/μg = particles/μg; p/mL = particles/mL

**Table S4. Distribution of the top 10 miRNAs in Ev1–Ev3**

| **miRNAs Ev1** | **UMI Ev1** | **Percent Ev1** | **miRNAs Ev2** | **UMI Ev2** | **Percent Ev2** | **miRNAs Ev3** | **UMI Ev3** | **Percent Ev3** |
| --- | --- | --- | --- | --- | --- | --- | --- | --- |
| hsa-miR-16-5p | 28668 | 16.4% | hsa-miR-16-5p | 43147 | 16.4% | hsa-miR-16-5p | 14689 | 13.4% |
| hsa-miR-146a-5p | 14818 | 8.5% | hsa-miR-146a-5p | 22841 | 8.7% | hsa-miR-146a-5p | 7578 | 6.9% |
| hsa-miR-126-3p | 11508 | 6.6% | hsa-miR-126-3p | 17320 | 6.6% | hsa-miR-126-3p | 7231 | 6.6% |
| hsa-let-7f-5p | 8736 | 5.0% | hsa-let-7f-5p | 13281 | 5.1% | hsa-let-7f-5p | 6894 | 6.3% |
| hsa-miR-223-3p | 5431 | 3.1% | hsa-miR-223-3p | 8167 | 3.1% | hsa-let-7a-5p | 3759 | 3.4% |
| hsa-let-7a-5p | 4992 | 2.9% | hsa-let-7a-5p | 7737 | 2.9% | hsa-miR-223-3p | 3534 | 3.2% |
| hsa-let-7i-5p | 4678 | 2.7% | hsa-let-7i-5p | 6919 | 2.6% | hsa-let-7i-5p | 3018 | 2.7% |
| hsa-miR-423-5p | 4507 | 2.6% | hsa-miR-423-5p | 5289 | 2.0% | hsa-let-7b-5p | 2284 | 2.1% |
| hsa-let-7b-5p | 3447 | 2.0% | hsa-miR-199b-3p | 5087 | 1.9% | hsa-miR-199b-3p | 2147 | 2.0% |
| hsa-miR-21-5p | 3377 | 1.9% | hsa-miR-21-5p | 5060 | 1.9% | hsa-miR-199a-3p | 2136 | 1.9% |

**Table S5. The expression profiles of microRNAs expressed in the six batches of UC-sEvs with CV%**

|  | miR-126-3P | | Average | miR-146a-5p | | Average | miR-16-5p | | Average | miR-223-3p | | Average | let-7i-5p | | Average | let-7a-5p | | Average |
| --- | --- | --- | --- | --- | --- | --- | --- | --- | --- | --- | --- | --- | --- | --- | --- | --- | --- | --- |
| 1 | 6.25* | 6.24 | 6.24 | 0.65 | 0.68 | 0.67 | 1.21 | 1.17 | 1.19 | 6.39 | 5.83 | 6.11 | 0.80 | 0.82 | 0.81 | 0.56 | 0.53 | 0.54 |
| 2 | 5.84 | 5.83 | 5.84 | 0.87 | 0.70 | 0.79 | 0.51 | 0.54 | 0.53 | 7.16 | 8.06 | 7.61 | 0.45 | 0.47 | 0.46 | 0.50 | 0.45 | 0.48 |
| 3 | 4.04 | 4.06 | 4.05 | 0.51 | 0.55 | 0.53 | 0.87 | 0.88 | 0.88 | 4.21 | 4.02 | 4.12 | 0.12 | 0.13 | 0.13 | 0.11 | 0.13 | 0.12 |
| 4 | 1.90 | 3.94 | 2.92 | 0.87 | 0.96 | 0.91 | 1.80 | 1.20 | 1.50 | 4.60 | 5.09 | 4.84 | 0.78 | 0.81 | 0.80 | 0.67 | 0.68 | 0.67 |
| 5 | 2.93 | 2.90 | 2.92 | 0.81 | 0.81 | 0.81 | 1.08 | 1.08 | 1.08 | 1.85 | 2.04 | 1.94 | 0.25 | 0.28 | 0.26 | 0.19 | 0.22 | 0.20 |
| 6 | 4.31 | 4.40 | 4.36 | 0.62 | 0.66 | 0.64 | 0.86 | 0.90 | 0.88 | 4.08 | 4.79 | 4.43 | 0.17 | 0.20 | 0.18 | 0.78 | 0.81 | 0.79 |
| STDEV |  | | 1.36 |  | | 0.14 |  | | 0.33 |  | | 1.92 |  | | 0.30 |  | | 0.26 |
| AVERAGE |  |  | 4.39 |  |  | 0.72 |  |  | 1.01 |  |  | 4.84 |  |  | 0.44 |  |  | 0.47 |
| CV% |  |  | 30.94 |  |  | 19.14 |  |  | 32.82 |  |  | 39.57 |  |  | 68.84 |  |  | 55.84 |

* = 2^-△ (miRNA CT- U6 CT)^

STDEV = standard deviation; CV = coefficient of variation; CV % =STDEV/AVERAGE × 100%.

**Supplementary figures and figure legends**

**
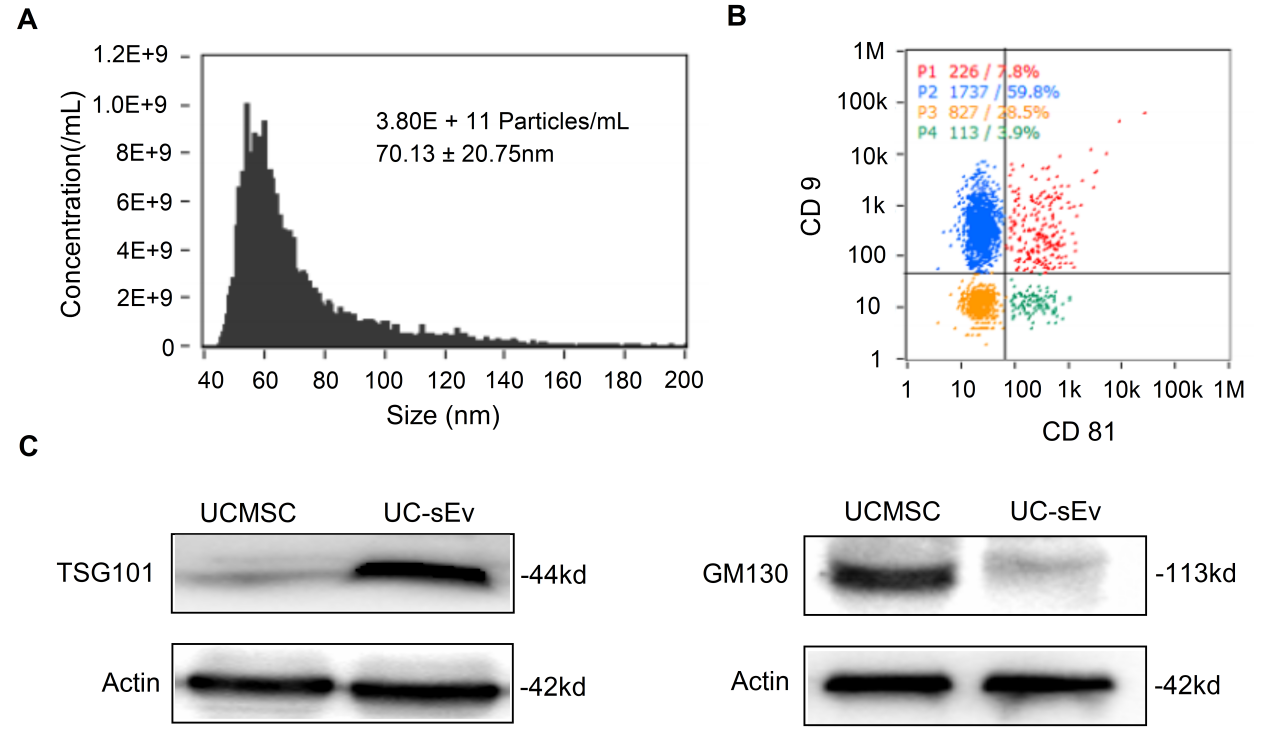
**

**Figure S1.** Detection data of UC-sEvs. (A) A histogram of particle size and concentration for a UC-sEV sample detected via nFCM. (B) Immunofluorescence staining for CD9 and CD81 detected via nFCM. (C) Immunoblots of a cell lysate and a UC-sEV preparation.

**
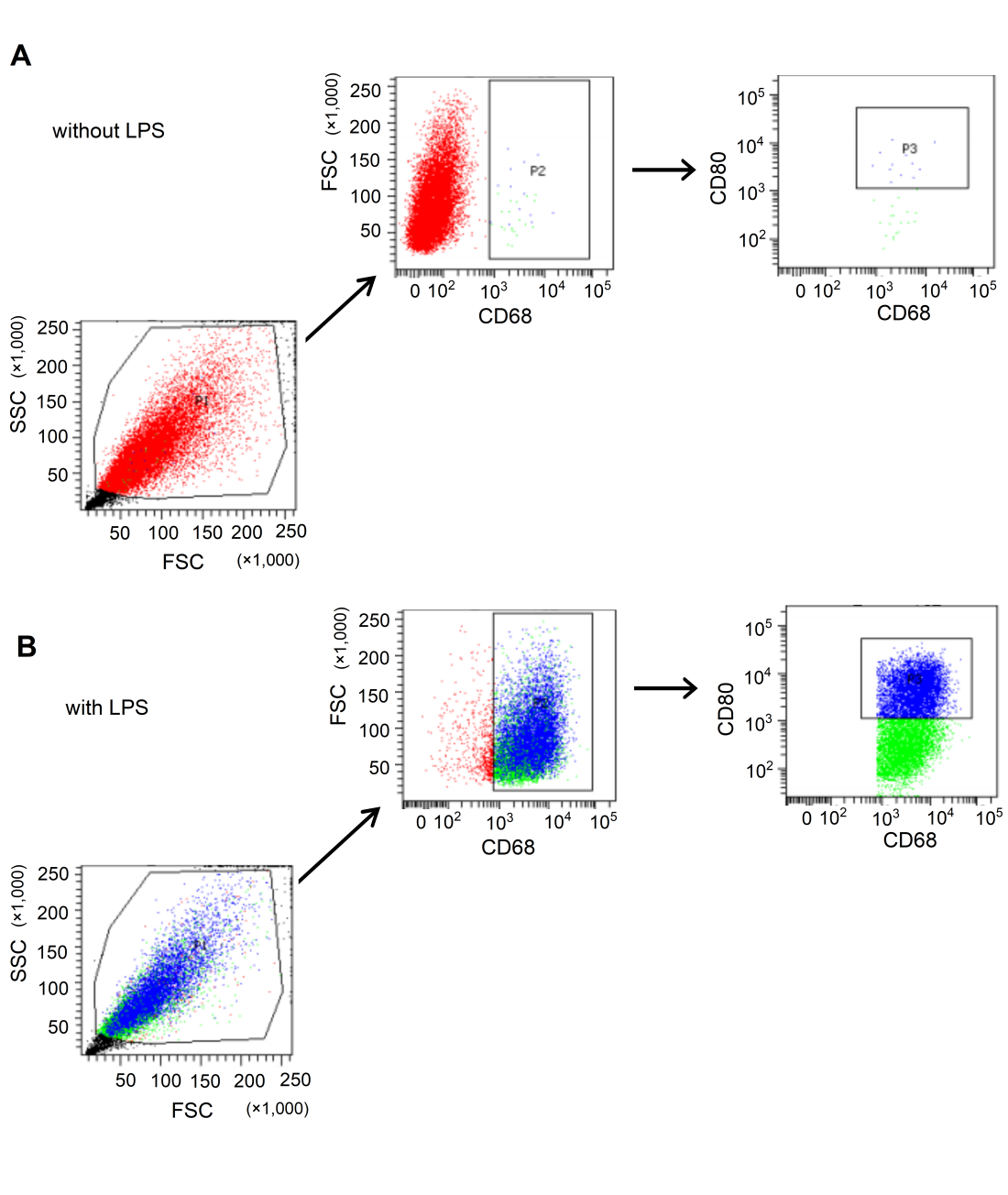
**

**Figure S2.** Gating strategy for the flow cytometry analyses of M1 macrophages in induced THP-1 cells, corresponding to Figure 2H in the main text.

**
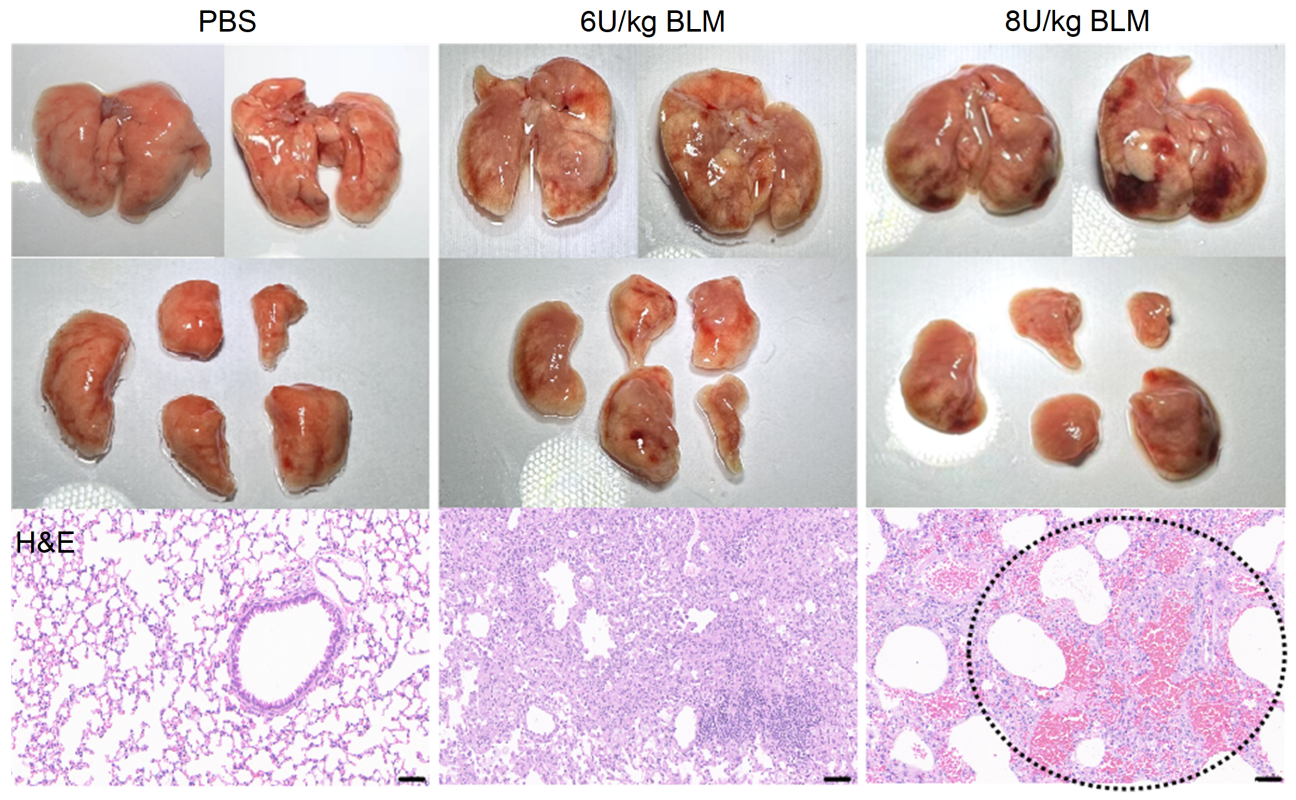
**

**Figure S3.** In vitro images of lungs (top and middle panels) and representative images of HE-stained lung sections (bottom panel). Bottom: Scale bar = 50 μm. Dashed circle: large area with focal bleeding.


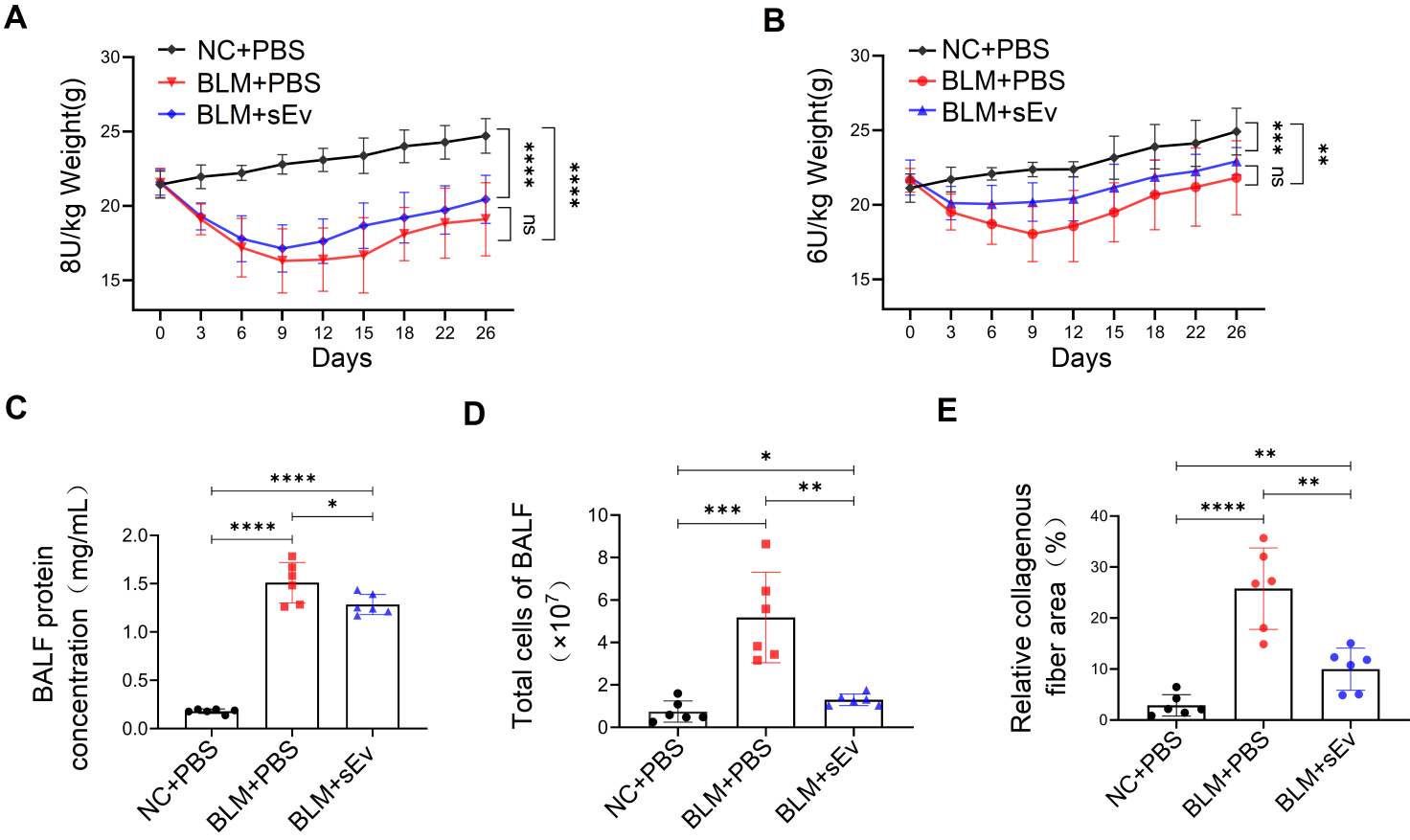


**Figure S4.** Comparison of body weights in IPF mice. Body weights of IPF mice exposed to 8 U/kg (A) or 6 U/kg (B). Total protein concentrations (C) and total cell counts (D) in the BALF. (E) Relative quantification of the area of collagenous fibres in the lungs, as indicated by Sirius red staining, corresponding to Figure 5A in the main text. n = 6 per group. Each symbol represents 1 mouse. The data are presented as the means ± SDs. *P < 0.05, **P < 0.01, ***P < 0.001, and ****P < 0.0001; NC = negative control.


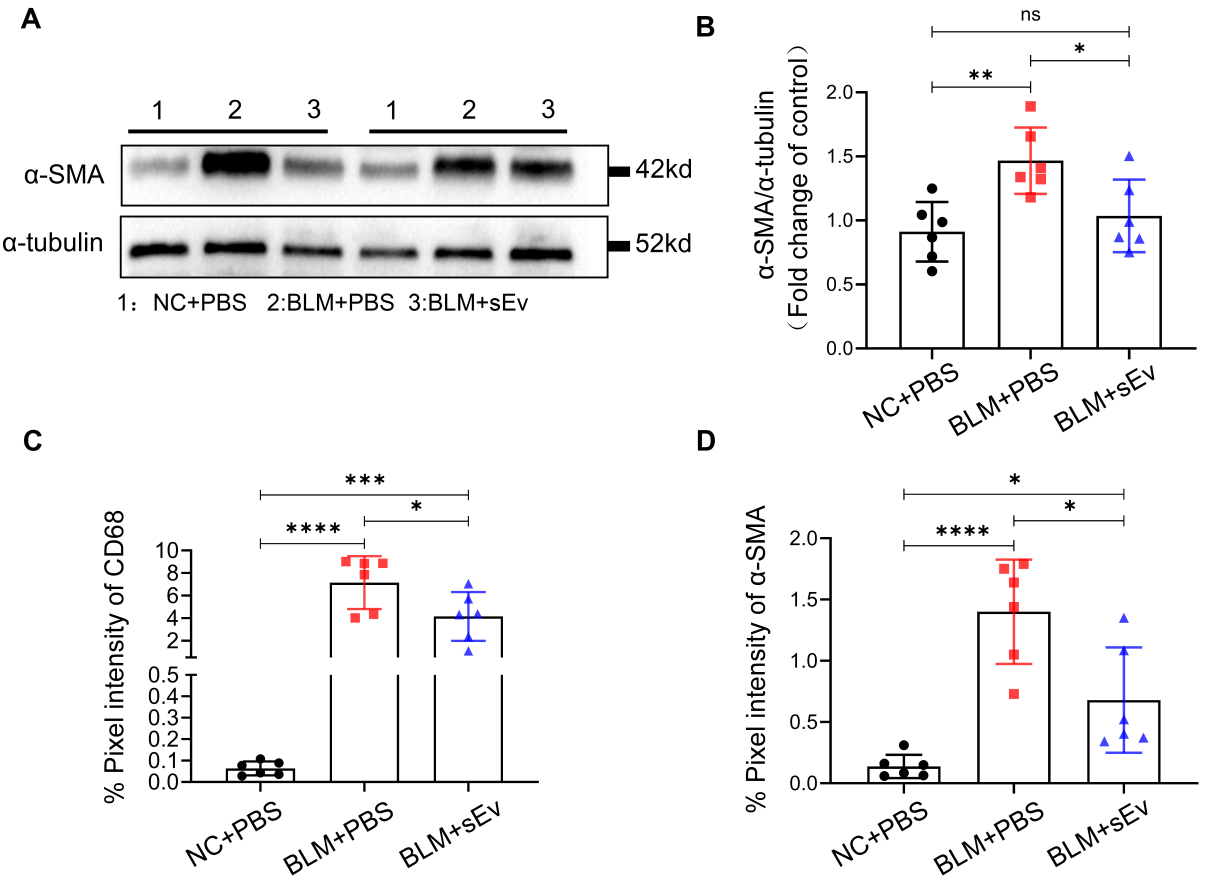


**Figure S5.** Fibrosis detection by western blotting. (A) Representative images of western blotting results of α-SMA expression in lung tissue lysates. α-Tubulin was used as a loading control. (B) Quantification of the ratio of α-SMA to α-tubulin. Quantitative analysis of fluorescence signals from CD68 (C) and α-SMA (D), corresponding to Figure 5D in the main text. *P < 0.05, **P < 0.01, ***P < 0.001, and ****P < 0.0001; NC = negative control. Analysis was performed via Image Pro Pius software; n = 6 per group. Each symbol represents 1 mouse.
