# Supplementary material for: Investigating the Therapeutic Efficacy of Quality-Controlled, miR-146a-5p-Enriched Small Extracellular Vesicles Derived From MSCs Against Idiopathic Pulmonary Fibrosis
Source: Stem Cell Rev Rep. 2025 Sep 24;22(1):523–44. doi: 10.1007/s12015-025-10976-8 (PMC12795917; doi:10.1007/s12015-025-10976-8)
Supplement: Supplementary file 2 — (DOCX 3.02 MB) [file 12015_2025_10976_MOESM2_ESM.docx]

**Supplementary File 2**

**Investigating the therapeutic efficacy of quality-controlled, miR-146a-5p-enriched small extracellular vesicles derived from MSCs against idiopathic pulmonary fibrosis**

Xin Wang ^1,*^ , Lingjiao Meng ^1,*^ , Qiuhong Wang^1^, Ruixue Rong^1^, Yu Zhang^3^, Xiaohui Zhao^1^, Chen Liang^1^, Huizhen Guo^1^, Li Deng^4^ , Zengqi Tan^5^, Feng Guan^6^ , Yi Tan ^1,2^

^1^Qilu Cell Therapy Technology Co., Ltd, Gangyuan 6th Road, Licheng District, Ji'nan, Shandong 250000, P. R. China.

^2^Shandong Yinfeng Life Science Research Institute, Ji'nan, P. R. China.

^3^Department of Otolaryngology, Head and Neck Surgery, Yantai Yuhuangding Hospital, Qingdao University, Yantai, P. R. China.

^4^Department of Gastroenterology, The First Affiliated Hospital of Shandong First Medical University & Shandong Provincial Qianfoshan Hospital, Ji'nan, P. R. China.

^5^School of Medicine, Northwest University, Xi'an, P. R. China.

^6^Key Laboratory of Resource Biology and Biotechnology in Western China, Ministry of Education, Provincial Key Laboratory of Biotechnology, College of Life Sciences, Northwest University, Xi'an, P. R. China.

* These authors have contributed equally to this work.

**Corresponding author:** Yi Tan

**Address:** Qilu Cell Therapy Technology Co., Ltd, Gangyuan 6th Road, Licheng District, Jinan, Shandong 250000, P. R. China.

**Tel:** +86-0531-88233100; **E-mail:** pkuty@126.com

**Original, full-length gel and blot images for Fig 7D**


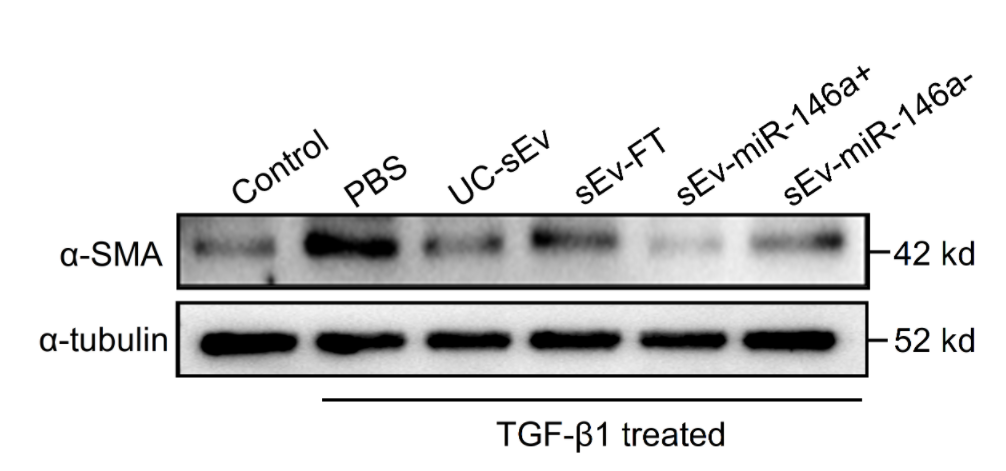


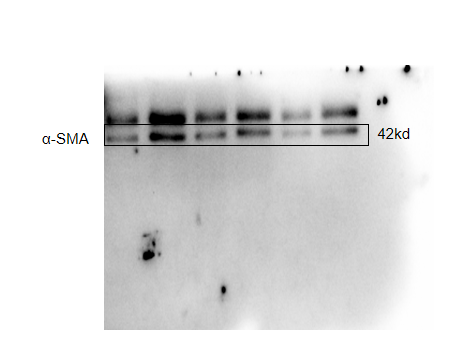


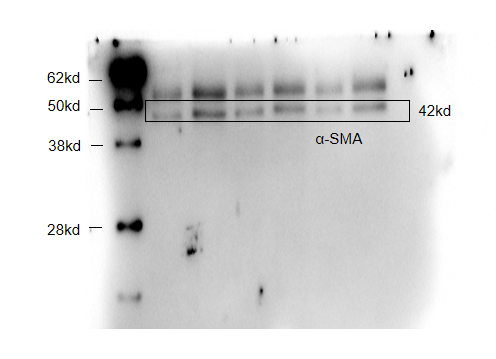


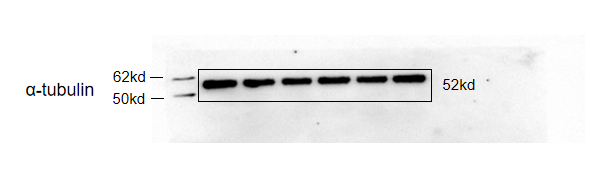


**Original, full-length gel and blot images for Fig S1.C**


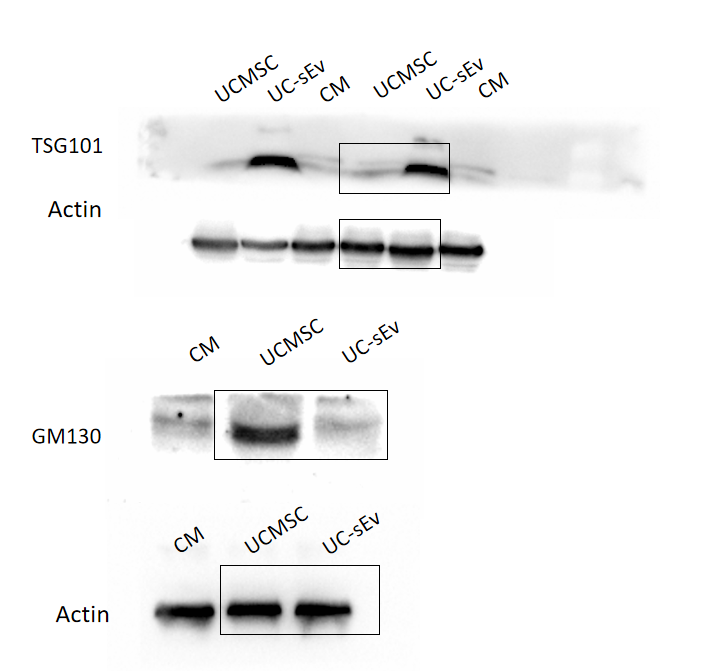


**
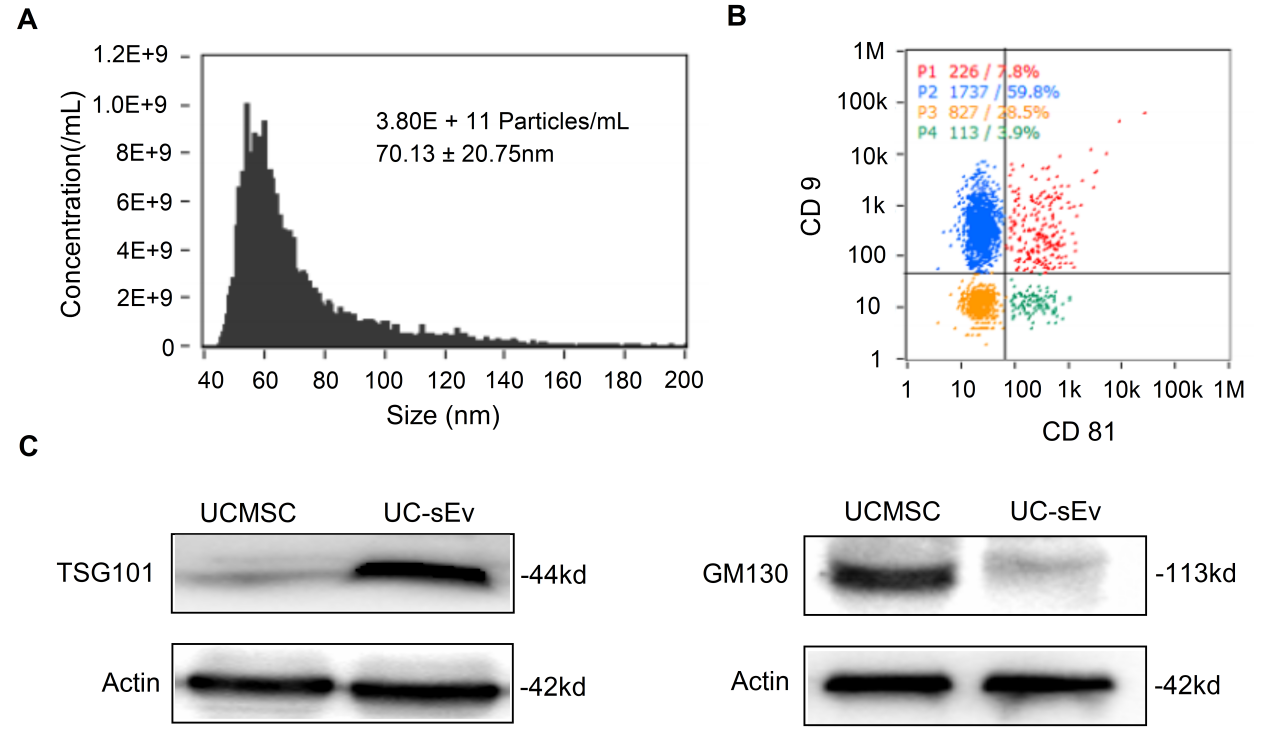
**

**Original, full-length gel and blot images for Fig S5.A**


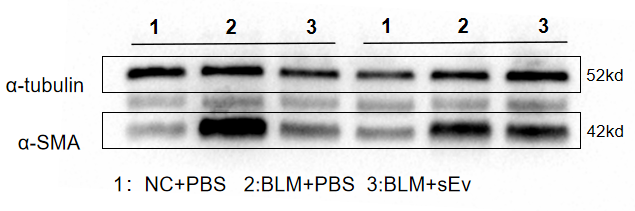


**
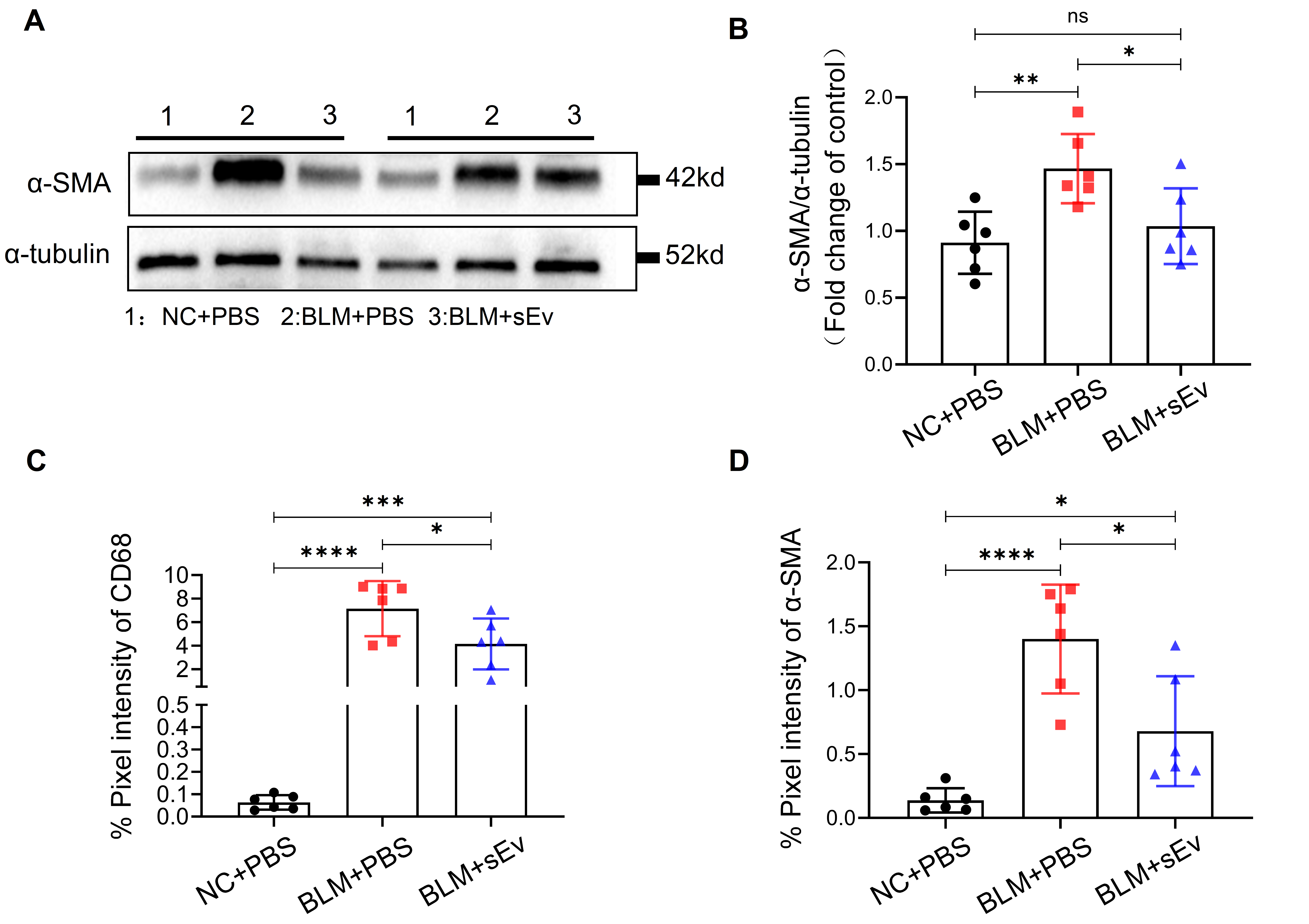
**
